# Targeting Tumor Cells with Anti-CD44 Antibody Triggers Macrophage-Mediated Immune Modulatory Effects in a Cancer Xenograft Model

**DOI:** 10.1371/journal.pone.0159716

**Published:** 2016-07-27

**Authors:** Daniela Maisel, Fabian Birzele, Edgar Voss, Adam Nopora, Sabine Bader, Thomas Friess, Bernhard Goller, Daphna Laifenfeld, Stefan Weigand, Valeria Runza

**Affiliations:** 1 Translational Technologies and Bioinformatics, Pharmaceutical Sciences, Roche Innovation Center Munich, Penzberg, Germany; 2 Discovery Oncology, Roche Innovation Center Munich, Penzberg, Germany; 3 Large Molecule Research, Roche Innovation Center Basel, Basel, Switzerland; 4 Selventa Inc., Cambridge, Massachusetts, 02140, United States of America; University of Patras, GREECE

## Abstract

CD44, a transmembrane receptor reported to be involved in various cellular functions, is overexpressed in several cancer types and supposed to be involved in the initiation, progression and prognosis of these cancers. Since the sequence of events following the blockage of the CD44-HA interaction has not yet been studied in detail, we profiled xenograft tumors by RNA Sequencing to elucidate the mode of action of the anti-CD44 antibody RG7356. Analysis of tumor and host gene-expression profiles led us to the hypothesis that treatment with RG7356 antibody leads to an activation of the immune system. Using cytokine measurements we further show that this activation involves the secretion of chemo-attractants necessary for the recruitment of immune cells (i.e. macrophages) to the tumor site. We finally provide evidence for antibody-dependent cellular phagocytosis (ADCP) of the malignant cells by macrophages.

## Introduction

CD44 is a transmembrane receptor reported to be involved in various cellular functions like adhesion, homing, migration and extravasation [1–3]. It is overexpressed in several cancer types [4–6], and there is also evidence that its expression is involved in the initiation, progression and prognosis of these cancers [7–11]. Although the CD44 protein is a rather simple transmembrane molecule (single-chain, single-pass transmembrane receptor), its complexity is the result of multiple isoforms [12,13], which arise by the alternative splicing of variant exons 1–10 (v1-v10). Exon v1 is not expressed in human cells since it contains a stop codon. Furthermore, CD44 isoforms are highly N- and O-glycosylated proteins [14,15]. Signaling through CD44 involves the formation of complexes with various receptors which have also been involved in cancer, such as cMET, EGFR, HER2 and VEGFR [16–19], as well as the interaction with its main natural ligand, hyaluronic acid (HA) [20,21]. We have recently shown that blocking CD44-binding to HA with RG7356, an anti-CD44 antibody directed against the constant region of CD44, prevents tumor cell adhesion *in vitro* and leads to tumor growth inhibition in several *in vivo* xenograft models [22]. Furthermore, in a previous global phospho-proteomic analysis we showed that treatment with RG7356 leads to a significant down-regulation of the MAPK cascade after 4.5 hours [23]. However, the detailed sequence of transcriptional changes and subsequent events following the blockage of the CD44-HA interaction has not yet been studied in detail.

Here, we aimed to elucidate the mode of action of RG7356 to understand the tumor microenvironment changes triggered by this antibody. For this purpose, we profiled xenograft tumors by RNASeq which allows assessment of treatment-specific effects on both human tumor and mouse stroma cells in a single expression profiling experiment. We show that RG7356 has an immune stimulatory effect whereas cytokine measurements suggest that this effect is mainly driven by macrophages. We further provide evidence that the immune-stimulatory nature of the molecule is FcγR-dependent and that antibody-dependent cellular phagocytosis (ADCP) constitutes its main mode of action.

## Results

### RNA sequencing reveals differences in immune response activation between the tumor and the host

Using a global phospho-proteomics analysis, we have previously shown that treatment with RG7356 triggers the modulation of the MAPK pathway in an MDA-MB-231 xenograft model, where RG7356 (1mg/kg) leads to 85% tumor growth inhibition (TGI) [23]. In a subsequent set of experiments we aimed to understand the extent of the observed phospho-proteomic changes and their relevance in differential gene expression. Interestingly, a combined gene-expression and phospho-proteomics analysis suggested an activation of the immune system in this immune-deficient (SCID/bg) xenograft model in response to treatment with RG7356 (S1 Materials and Methods). In order to test if the observed immune response involves only the host’s stroma and the limited immune cell infiltrate, or also the tumor cell line of human origin, an analysis of both human and mouse immune modulators is necessary. This would additionally shed some light on the underlying mechanisms leading to the activation of an overall immune response and the interplay between tumor and host. On the other hand, since RG7356 is not mouse cross-reactive, the secretion of murine cytokines cannot be initiated by direct binding of RG7356 to CD44 on immune cells but can be either FcγR-mediated and/or indirectly triggered by tumor-derived immune modulators. With this in mind, we chose an experimental setup including an analysis pipeline enabling the measurement of both the tumor’s and the host’s gene-expression profile at the same time (S1 Fig).

**Fig 1:**
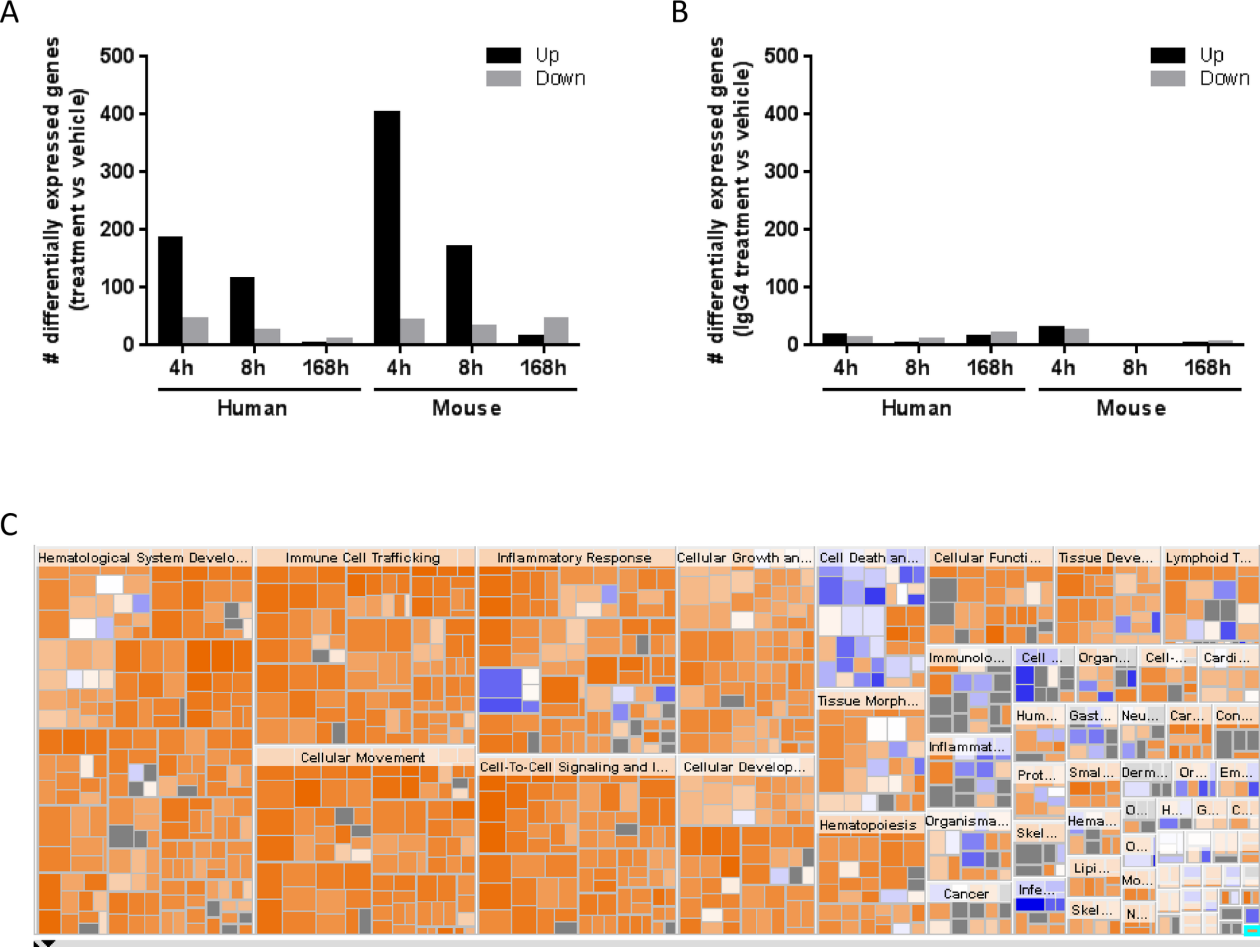
RG7356 treatment leads to a deregulation of genes in both tumor and host at early time points. (A) Number of differentially expressed human and mouse transcripts at 3 different time points of anti-CD44 treatment bearing the IgG1 backbone compared to vehicle control. (B) Number of differentially expressed human and mouse transcripts at 3 different time points of anti-CD44 treatment bearing the IgG4 backbone compared to vehicle control. (C) Heatmap view of predicted activity of biological processes obtained from IPA® downstream processes analysis. Activated processes are shown in different shades of orange, inhibited processes are shown in different shades of blue. If no activity assessment was possible, the biological process is shown in grey.

In brief, MDA-MB231 xenografts were grown for 64 days until median tumor volume reached approximately 250 mm^3^ before treatment with RG7356 was started. In order to elucidate the role of the Fc moiety of RG7356, which–as an IgG1 –bears immune effector functions, an “Fc-silent” version of this antibody was also included in the study, in which RG7356 was expressed as IgG4. Whole tumors were then excised at 4, 8 and 168 hours post-treatment, RNA was extracted and sequenced using Illumina RNA sequencing (RNASeq) which, in contrast to array platforms, allows the measurement of mouse and human RNA patterns in a single experiment. The detailed RNAseq data analysis workflow can be found in Material and Methods. Briefly, reads were aligned against the mouse and human transcriptomes and reads that mapped to both transcriptomes were discarded from further analysis. Gene expression levels (RPKMs) were computed as proposed by Mortazavi *et al* [24] and DESeq [25] was then used to identify differentially regulated genes between the conditions of interest. As depicted in Fig 1A, we observed time-course-dependent human gene-expression profiles with a high number of acute changes occurring at early time points and almost no significant changes 168 hours post-treatment (Fig 1A, left panel). This pattern was also observed for the murine genes with about twice as many differentially expressed transcripts detected (Fig 1A, right panel). In contrast, almost no significantly differentially expressed genes were observed after treatment with the IgG4 antibody (Fig 1B, S5 and S6 Tables).

Focusing on these fast and transient events, we used QIAGEN’s Ingenuity® Pathway Analysis (IPA) [26] to perform a functional characterization of the observed changes at 4h and 8h. Using IPA’s Downstream effects analysis we observed that in both, the tumor and the host, the same biological processes and functions were affected by RG7356. These included–but were not limited to–an inflammatory response, cellular movement and immune cell trafficking (S2 Fig, left panel) and were all predicted to be strongly activated (Fig 1B and S2 Fig, right panel). Furthermore, enrichment analysis of canonical pathways revealed that, in both the tumor and the host, the most highly enriched pathways are connected to an inflammatory response (S3 Fig).

When ranking the modulated processes based on their activation scores, it seems likely that the tumor response is driven by the activation, migration and differentiation of immune cells (Table 1, full list in S1 Table), while the host’s response is mainly driven by cell recruitment and survival (Table 2, full list in S2 Table).

**Table 1:** Tumor responses to RG7356 involve activation, migration and differentiation of immune cells.

Diseases or Functions Annotation	p-Value	Predicted Activation State	Activation z-score
cell movement	5,07E-18	Increased	4,882
migration of cells	3,98E-17	Increased	4,493
activation of blood cells	2,48E-20	Increased	4,098
activation of cells	1,16E-21	Increased	3,951
activation of leukocytes	3,86E-20	Increased	3,935
differentiation of cells	1,13E-12	Increased	3,877
migration of mononuclear leukocytes	8,88E-14	Increased	3,794
activation of phagocytes	1,37E-14	Increased	3,785
cellular homeostasis	5,37E-13	Increased	3,767

10 most significantly modulated processes resulting from IPA’s Downstream effects analysis, sorted by activation z-score.

**Table 2:** Host responses to RG7356 involve recruitment of immune cells and cell survival.

Diseases or Functions Annotation	p-Value	Predicted Activation State	Activation z-score
cell survival	1,03E-12	Increased	4,525
cell viability	2,00E-12	Increased	4,306
recruitment of blood cells	6,64E-23	Increased	3,889
recruitment of leukocytes	3,97E-22	Increased	3,776
migration of granulocytes	3,34E-16	Increased	3,775
recruitment of granulocytes	5,20E-19	Increased	3,460
recruitment of phagocytes	1,73E-20	Increased	3,443
migration of neutrophils	3,75E-15	Increased	3,402
migration of cells	1,41E-25	Increased	3,399

10 most significantly modulated processes resulting from IPA’s Downstream effects analysis, sorted by activation z-score.

In addition, the tumor’s and the host’s affected pathways also differentiate on the gene level, which suggests that despite being involved in the same biological processes a differential set of genes is responsible in activating the biological answer in either the tumor or the host (S4 Fig). The lists of genes driving the predicted activation of the immune response can be found in S3 and S4 Tables.

**Fig 2:**
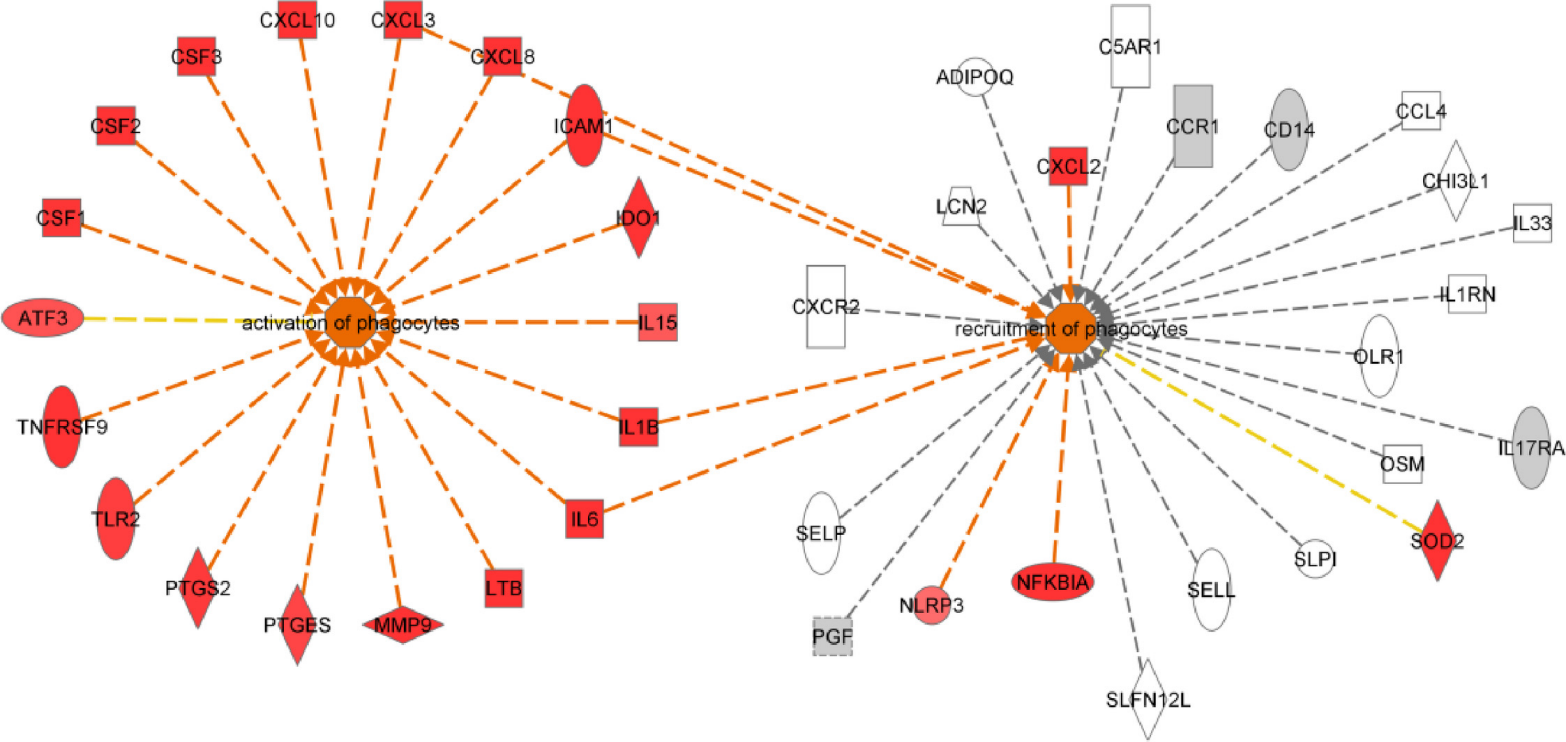
**Tumor and host responses to RG7356 are driven by a different set of genes** Network view of two activated processes in the tumor (left) and the host (right). The overlaid gene expression values are Fold Changes derived from the tumor analysis.

For the tumor (S3 Table), an up-regulation of NFκB target genes was observed (e.g. IL-6, IL-8, ICAM1, S5 Fig) as well as of cytokines typically expressed by epithelial cells or fibroblasts involved in the first events of an immune response. In particular, an up-regulation of genes related to the activation of macrophages (e.g. CSF-1, CSF-2, CSF-3, CXCL10, IL-8, IL-1β and IL-15) was detected. On the other hand, amongst the modulated murine genes, deregulation of cytokines and receptors secreted and expressed by different types of immune cells (e.g. CCL2, CCR1, CCRL2, CXCL1 and CXCL6) were observed, pointing to second-line events of an immune response. In addition, an up-regulation of macrophage receptors involved in cell activation and recruitment (e.g. CD80, CD86, CSF3R, CSF2RB and CD209) was observed 8 hours after treatment (S4 Table) possibly indicating an increased presence of activated macrophages in the tumor at this point in time. The difference between deregulation of genes in the tumor and the host can be exemplified by two processes involving phagocytes (Fig 2B): while activation of phagocytes is seen in the tumor (left), recruitment of phagocytes is stimulated in the host (right). An overlay of the deregulated genes in the tumor (red genes) shows almost no overlap with the genes deregulated in the host.

Based on these findings, as well as the fact that macrophages are described to be the main immune cell type in SCID/bg mice reacting to xenotransplantations [27], we hypothesized that treatment with RG7356 antibody leads to a macrophage-mediated activation of the immune system.

### Cytokine release profiling shows clear pattern of tumor- and host-mediated effects

Based on the results from the mRNA data analysis, a major difference between cytokines released by either the host or the tumor is expected. In order to test this on the protein level, a Luminex assay was performed using two cytokine panels, representing the human tumor and the murine host. These panels included 29 and 27 cytokines, respectively with 22 overlapping analytes (S7 Table). Because RNASeq data suggested that the prominent acute changes decline after 168 hours, we compared early (8 hours) and late (168 hours) events (S1 Fig).

**Fig 3:**
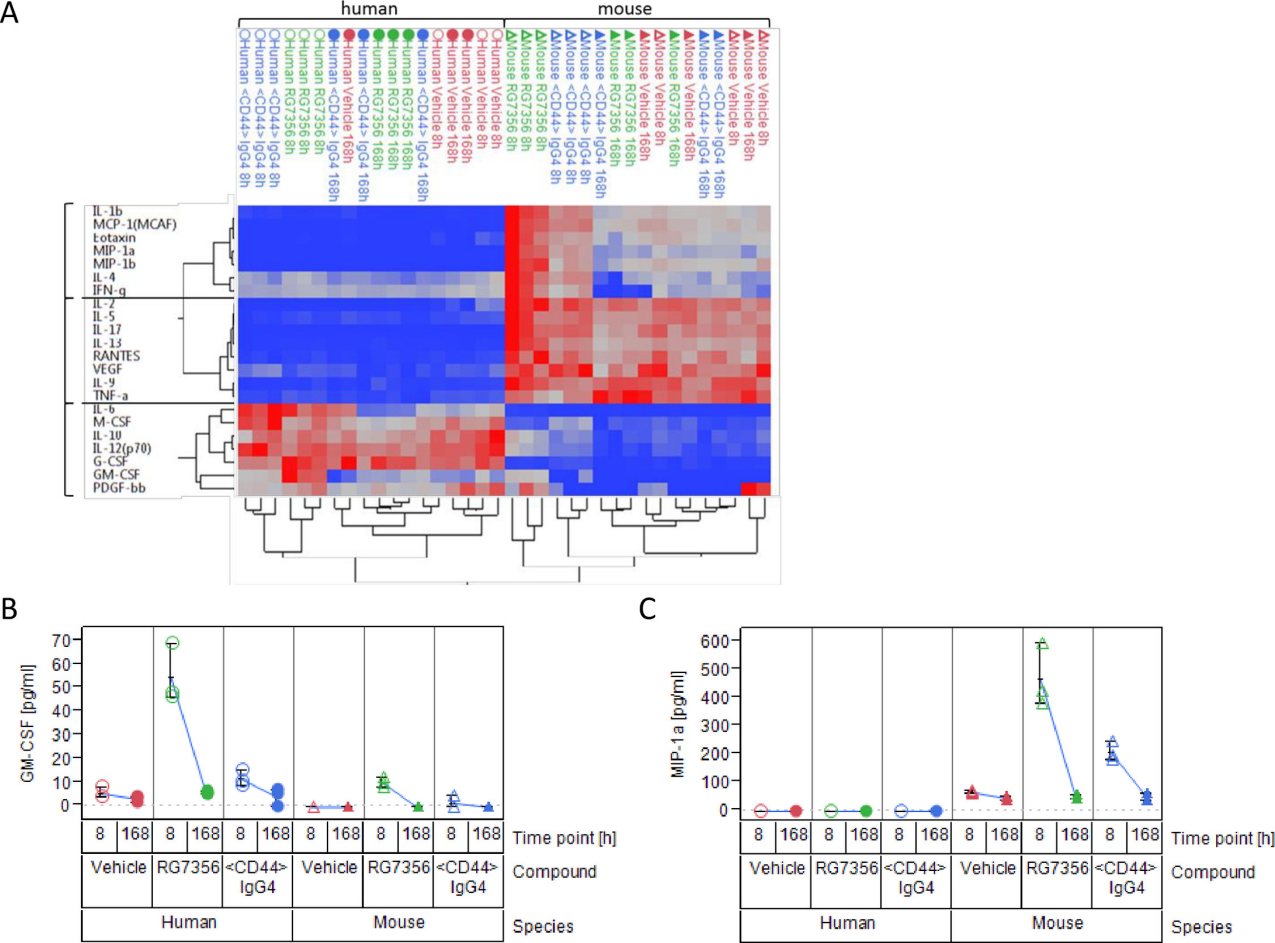
Tumor and host samples show distinct cytokine secretion profiles upon RG7356 treatment. (A) Cluster analysis of Luminex cytokine panel. Samples were analyzed at 8 and 168 hours after treatment in both human- and mouse-specific assays. Rows represent analytes, columns represent samples. Only analytes covered by both panels were used for clustering. Circles represent human samples, triangles represent mouse samples; open and solid symbols represent 8 and 168 hours of treatment, respectively, while color codes depict the different treatment groups. (B) and (C) Side-by-side comparison of human (left) and mouse (right) analyte concentrations (pg/ml) for the three different treatments. Shown are concentrations for GM-CSF (B) and MIP-1a (C).

The obtained data was clustered and, in accordance to previous findings, tumor and host show a distinct cytokine pattern where deregulation of cytokine expression occurs early in time followed by a decline to baseline levels at the later time point (Fig 3A). Further statistical analysis identified significant effects associated to the factors species, compound and time (S8 Table). All analytes that were measured in both human and mouse, showed a significant difference between species, except for PDGF-bb. Moreover, ten of these analytes also showed a significant difference between compounds and over time, originating mostly from a deregulation in one of the species. The main changes occurring in the tumor involve an up-regulation of GM-CSF, IL-8 and IP-10 (the latter two only measured for human; S6 Fig), which are known specific chemo attractants for immune cells, e.g. macrophages [28–30]. On the other hand, upon treatment a different set of cytokines is preferentially up-regulated by the host, such as IL-1β, MCP-1, Eotaxin, MIP-1a, MIP-1b, IL-4, IL-13, IFN-γ, IL-3 and KC (the latter two only measured for mouse; S7 Fig). These cytokines are typically secreted by monocytes and macrophages upon stimulation [31] and are crucial for mounting an effective innate immune response [32]. Fig 3 provides examples of two modulated cytokines for either the tumor (GM-CSF, Fig 3B) or the host (MIP-1a, Fig 3C), which are increased specifically upon treatment with RG7356, as effector-function competent IgG1, and not by the IgG4.

**Fig 4:**
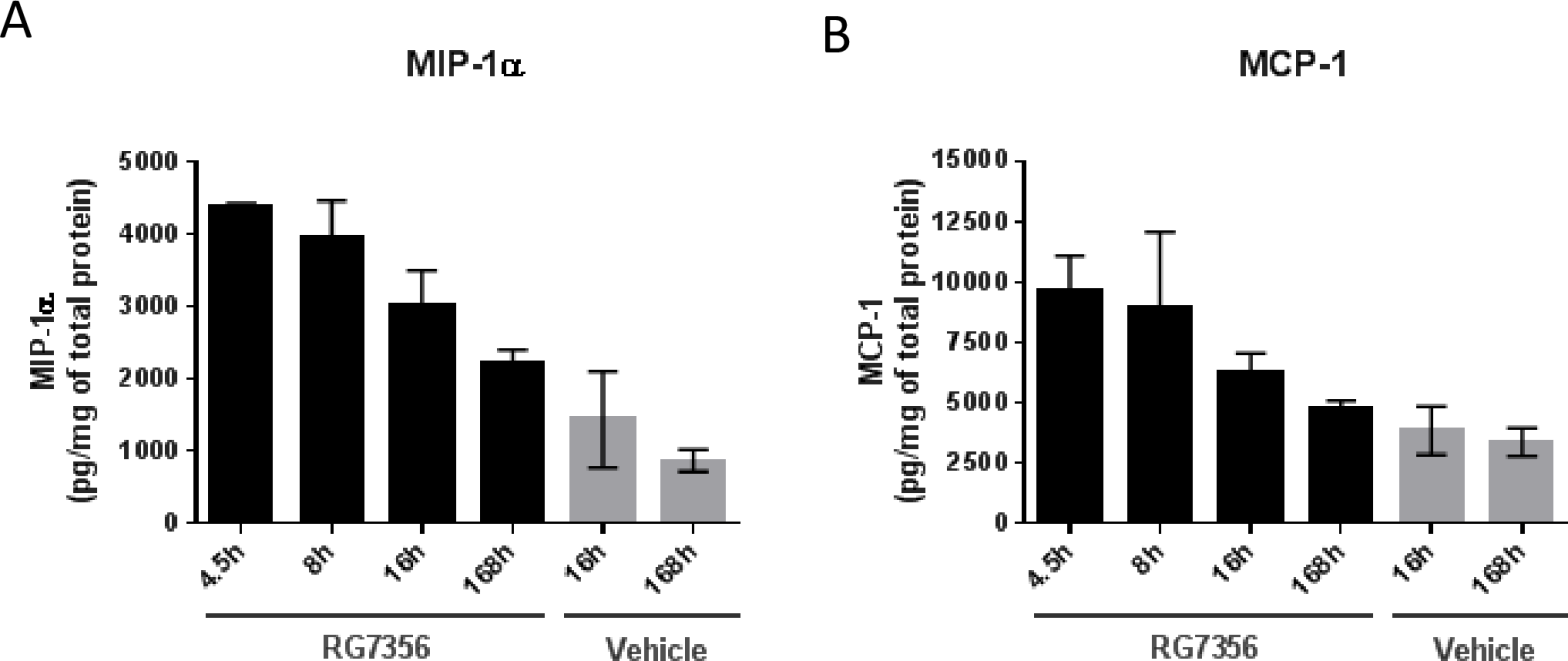
The increased amounts of MIP-1a and MCP-1 decline to baseline levels after one week. Time-course study of MDA-MB231 xenografts over one week after treatment with RG7356. MIP-1a (A) and MCP-1 (B) concentrations were analyzed in obtained tumor lysates by ELISA.

In addition, the pharmacodynamics of MIP-1a and MCP-1, two molecules especially secreted by macrophages [33] was quantitatively monitored by ELISA in a time-course experiment. As depicted in Fig 4A and 4B, analytes were up-regulated at 4.5 hours of treatment and steadily decreased over time to control levels and therefore confirmed the results obtained from both RNASeq and Luminex analyses.

### RG7356 leads to phagocytosis of tumor cells by macrophages

**Fig 5:**
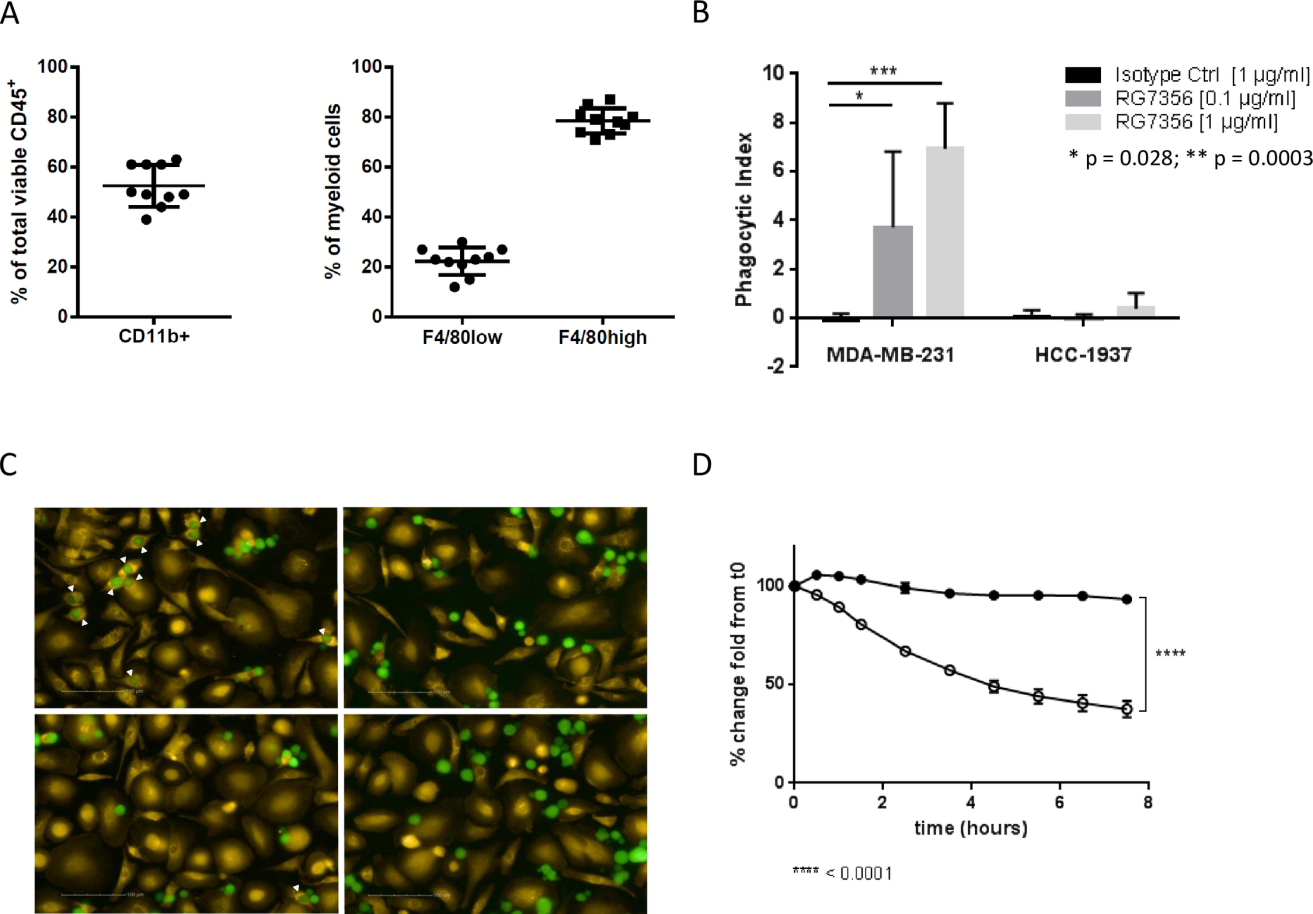
RG7356 treatment leads to phagocytosis of tumor cells. (A) *Ex vivo* analysis of MDA-MB-231 xenograft immune infiltrates. Left panel: percentage of CD45^+^CD11b^+^ myeloid cells in total infiltrate. Right panel: tumor-associated macrophages (F4/80^high^) and myeloid-derived suppressor cells (F4/80^low^) as percentage of the myeloid compartment. (B) RG7356-opsonized MDA-MB-231 and HCC-1937 cells were pre-incubated with macrophages and phagocytosis was assessed by flow cytometry after 30 min as described in Material and Methods. Results represent the outcome of 3 independent experiments. (C) MDA-MB-231 (upper panels) and HCC-1937 (lower panels) cells were pre-incubated with RG7356 (left panels) or isotype control (right panels) and cultured together with macrophages, as described in Materials and Methods. Pictures were taken every 30 min for 10 hours (see S1 and S2 Videos). Pictures shown here correspond to the 0.5h time point and are representative of at least 3 independent experiments. Arrowheads indicate engulfed tumor cells. Macrophages are shown in yellow, tumor cells in green. (D) Time-course quantification of total tumor cells (CFMDA signal) in MDA-MB-231/macrophages co-cultures in the presence of RG7356 (open circles) or isotype control (filled circles).

Since the RNA profiling data and the cytokine measurements suggested that macrophages might be involved in RG7356’s mode of action, we next performed an *ex vivo* analysis of the tumor infiltrate by flow cytometry. As shown in Fig 5A, although MDA-MB-231 are not heavily infiltrated tumors (~3% tumor infiltrate; data not shown), most of the viable immune cells present in the tumor microenvironment are indeed tumor-associated macrophages (TAMs), defined as CD11b^+^F4/80^high^ cells.

Therefore, assuming that these particular cells are the real key players of the immune response against the tumor upon RG7356 treatment, we aimed to functionally characterize their role by conducting a series of phagocytosis assays. For this, bone marrow progenitor cells were isolated from healthy mice, differentiated into macrophages *in vitro* and subsequently co-cultured with fluorescently-labeled MDA-MB-231 or HCC-1937 cells in the presence or absence of RG7356 (at two different concentrations) or IgG isotype control. The HCC-1937 cell line was chosen as a negative control since it is also derived from breast malignant tissue but, when grown as a xenograft and unlike MDA-MB-231, shows no response to anti-CD44 treatment (18% TGI; S8 Fig). The macrophage phagocytic activity was assessed by flow cytometry and a phagocytic index was calculated as described in Material and Methods.

As shown in Fig 5B, phagocytosis of MDA-MB-231 cells in the presence of RG7356 already occurs after 30 min in a dose-dependent manner. Furthermore, in order to visualize and confirm the phagocytic activity of macrophages in the presence of RG7356-opsonized tumor cells, live imaging was performed. In this case, the same two cell lines were CMFDA-labeled and incubated with RG7356 or an irrelevant IgG, and further cultivated together with CMTMR orange-labeled macrophages, as described in more detail in Materials and Methods. Fig 5C and S1 and S2 Videos clearly show that tumor cells are indeed engulfed by macrophages and not just attached to their membranes. Moreover, and as expected, only MDA-MB-231 cells pre-incubated with RG7356 are phagocytosed (Fig 5C, upper left panel) while neither the isotype control (right panels) nor RG7356 on the non-responder HCC-1937 cell line (lower left panel) led to phagocytosis. In addition, the specific decrease in the total number of CFMDA-positive cells at 7.5 hrs confirms that tumor cells are indeed depleted from the co-cultures (Fig 5D). No phagocytosis was seen when using the anti-CD44 IgG4 antibody or an irrelevant but binding IgG1 antibody (i.e. anti-CD45) (data not shown).

## Discussion

In this paper, we have analyzed the biological processes involved in the acute response to treatment with RG7356, an anti-CD44 antibody that binds to the constant region of CD44. Together with our previous findings [23], this work provides further insights into the cascade of events upon treatment with RG7356. Recent reports regarding anti-CD44 therapies mostly cover either inflammatory diseases and diabetes [34,35], or rather concentrate on the clinical aspects of oncological treatments [36]. Here, we provide evidence that one of the main cellular events following CD44-targeting results in macrophage-mediated phagocytosis of tumor cells. Using RNA sequencing and protein measurements of both tumor and host, we initially hypothesized that treatment with RG7356 not only affects the CD44-dependent tumor biology [23], but also modulates the malignant microenvironment by up-regulating cytokines and chemo-attractants responsible for the activation and recruitment, respectively, of tumor-associated macrophages (TAMs). Furthermore, these RG7356-specific effects seem to be on the one hand FcγR-dependent (since using the effector-function silent IgG4 version of the same antibody abolishes the immune modulatory reactions), and on the other hand are CD44-specific since an anti-CD45 binding IgG1 does not lead to phagocytosis (data not shown). Although TAMs are known to promote tumor progression [37], it has recently been shown that they maintain Fc-dependent anti-tumor functions and are able to phagocytose tumor cells in the presence of monoclonal antibodies targeting specific tumor antigens [38]. RG7356, therefore, belongs to such a group of antibodies that are able to influence the characteristic plasticity of TAMs in order to unmask their anti-tumor potential.

In Weigand *et al*. [23], we have shown that treatment with RG7356 results in the down-regulation of the MAPK pathway at 4.5h after treatment, which seems contradicting to the activation and initiation of an immune response that we observed in the present gene-expression study. However, we have analyzed further phospho-proteomics data of very early time points after treatment (0.5h and 1.5h, S1 Materials and Methods). Here, an activation of the MAPK cascade can be observed which results into an activation of the immune response observed at 4h and 8h in the tumor and the microenvironmet. The following down-regulation of signaling events as presented in Weigand et al. [23] then translate into a receding immune response and within a week, all levels are back to normal.

The reasons why the HCC-1937 cell line is not responsive to treatment with RG7356 remain unclear. Very recently, we have shown that there is a strong correlation between the predominant CD44 isoform and RG7356-mediated tumor growth inhibition [22], where the co-expression of any variable exon (“CD44v”) on a tumor cell is predictive for unresponsiveness, probably due to favorable cellular gained functions that arise from CD44v interactions *in cis* with other survival relevant receptors. For example, it has been shown that CD44v is able regulate the redox status in cancer cells by stabilizing the xCT protein (a glutamate-cystine transporter) which, in turn, promotes tumor growth [39]. Therefore, it is conceivable that, in this kind of cases, the tumor cells rely on this new gained function for survival and inhibiting the CD44-HA interaction remains biologically less relevant. Furthermore, HCC-1937 cells not only do carry the CD44v isoform but, in addition, secrete significantly low levels of hyaluronic acid, which is also associated to the isoform status and efficacy to treatment [22]. These observations together with the fact that HA is described as a strong activator of the immune response [40–42] might explain the present observations.

In summary, taken together these data indicate that upon binding to CD44^+^ tumor cells *in vivo* RG7356 triggers the secretion of chemo-attractants that are necessary for the recruitment of immune cells (i.e. macrophages) to the tumor site, finally leading to antibody-dependent cellular phagocytosis (ADCP) of the malignant cells by macrophages. These RG7356-specific effects are Fc-receptor dependent and seem to take place very early during treatment as they recede to baseline levels within a week.

## Materials and Methods

### Cell lines and culture

MDA-MB-231 cells (ATCC) were grown in DMEM supplemented with 2mM L-Glutamine, 10% heat-inactivated FBS and 1mM sodiumpyruvate. HCC-1937 cell line (DSMZ) was grown in RPMI-1640 without phenol-red supplemented with 10% heat-inactivated FCS + 2mM L-glutamin +penicillin/streptomycin. All media and supplements were purchased from PAN Biotech (Aidenbach, Germany). All cell lines were authenticated by the providers and cultivated for periods no longer than 6 months or re-authenticated by the PCR-based STR method.

### Xenograft studies

Immunodeficient SCID/bg mice were purchased from Charles River (Sulzfeld, Germany). Animals used in experiments were between 8 and 16 weeks of age. All animal experiments were approved by the Government of Upper Bavaria (permit number 55.2-1-54-2532.2-26-09) and performed according to the Directive 2010/63/EU of the European Parliament and of the Council on the protection of animals used for scientific purposes. Experimental techniques including humane endpoints were described in detail under this permit and all procedures were following strictly these definitions. In vitro passage number 4–5 were used for inoculation. 0.5 x 10^7^ MDA-MB-231 cells, were injected subcutaneously into the right flank of mice. At day 64 after tumor cell inoculation, when tumors were 234mm^3^ in median, mice were randomized and allocated to study groups. Therapeutic compounds or vehicle (20mM Histidine, 150mM NaCl, 0.01% Tween 80) were administered intraperitoneally at the indicated doses. Tumor volume (TV) was measured and calculated according to the NCI protocol (TV = (length x width^2^) / 2), where length and width are long and short diameters of tumor mass in mm^3^. At necropsy, tumors were excised and immediately shock frozen in liquid nitrogen.

### RNA sequencing

Total RNA was purified from fresh-frozen Mouse xenograft tumor samples by RNeasy Mini Kit (QIAGEN Cat. No. 74104, 74106). In brief, the entire sample was homogenized in RLT buffer using the Tissue Lyser (QIAGEN). After homogenization total RNA was extracted by following the RNeasy Mini Kit standard protocol. The quantity and quality of the RNA was analyzed using the NanoDrop Spectrophotometer and the Labchip Gx, respectively. For sequencing library preparation 1μg total RNA was used for the Illumina TruSeq RNA Sample Preparation Kits v2 (Illumina Cat. No RS-122-2001/2) by following the manufactures instructions. In brief, Poly-A RNA was purified and fragmented followed by first and second strand cDNA synthesis. After end repair and adenylation the Illumina adapters were ligated and the libraries were PCR amplified. Libraries were then quantified by SYBR Green (Kapa Biosystems, KK4602) PCR using Illumina specified oligos: 1.1–5’-AATGATACGGCGACCACCGAGAT-3’ and 2.1 5’- CAAGCAGAAGACGGCATACGA -3’. The PCR was run using the cycling protocol: 2 min at 95C then 40 cycles of 15 seconds at 95C and 30 seconds at 60C. After individual library quantification and dilution in Tris-Cl (10mM, pH 8.5) with 0.1% Tween 20 libraries were randomized and mixed in equimolar amounts. Libraries were clustered using the TruSeq PE Cluster Kit v3 on the c-bot (Illumina). After clustering the samples were sequenced on the HiSeq2000 (Illumina). Sequencing was done as 50 nucleotide paired-end yielding on average 30 Mio. reads per sample (min. 25 Mio., max. 35 Mio. reads).

### Statistics and bioinformatics analysis

#### RNASeq data analysis

Despite generally higher sensitivity and the possibility to analyze alternative splicing, the big advantage of using RNASeq over chip platforms in the xenograft setting is the fact that both species can be profiled at the same time in one single experiment (compared to two chip experiments that would have to be performed). Further, possible cross-mapping and cross-hybridization effects (in the chip setting) can be efficiently removed by RNASeq data analysis whereas for Affymetrix analyses they are much harder to identify and interpret. All RNASeq samples passed quality control in terms of number of reads per sample and read quality. Short reads were aligned to the human and mouse transcriptome (based on Ensembl v60) using Bowtie2 [43]. Based on the alignment we identified reads mapping to both transcriptomes at the same time and discarded them from further analysis. On average we found 70% of the reads to map to either transcriptome, with 7% of the reads mapping to both organisms which were subsequently filtered out. Across all samples, about 80% of the reads mapped to human, while 20% mapped to mouse which likely represents the quantity of human tumor cells compared to mouse stroma and immune cells. We then computed RPKM values for each gene as described by Mortazavi *et al*. [24] using the genes composite length, i.e. the sum of the length of all non-overlapping exon groups, as normalization factor using in-house tools. In order to identify differentially regulated genes we used the DESeq software package [25].

#### Pathway and biological function analysis

For pathway analysis purposes, we used Ingenuity Pathway Analysis (IPA). Built-in Fisher’s Exact Test was used for enrichment analysis of canonical pathways, Downstream Effects Analysis (DEA) was used for prediction of activation of biological processes. DEA was used as described in [44].

#### Luminex data analysis

The analysis of the Luminex data was initiated with a cluster analysis for descriptive analysis of the data using Ward’s method. Additionally, a statistical linear model including the factors compound, time, species and all pairwise interactions was fit to the data of each analyte. The benefit of this method is the potential to assess the effects of all factors on the data simultaneously. Of main interest were significant effects of species, compound and time; interactions of these factors gave additional information on the behavior of a specific analyte. All analyses were performed using the software JMP (SAS Institute Inc., Version 10.0).

### Luminex cytokine panel

For the analysis of the cytokine expression pattern and change during treatment MDA-MB-231 tumor xenograft samples were collected 8 and 168 hours after single treatment with RG7356, <CD44> IgG4 or vehicle. Lysates were generated and measured for human and mouse cytokines using commercial available cytokine multiplex assay kits from BioRad (BioPlex Mouse Cytokine Assay and Bio-Plex Human Cytokine Assay). Tests were done according to manufacturer’s protocol using a BioPlex 200. In total, 29 human and 27 mouse cytokines of interest were evaluated with 22 overlapping analytes.

### ELISA assay with tumor lysates

Excised and weighed tumors were mechanically homogenized in liquid nitrogen using mortar and pestle. After homogenization and prior to thawing of the powdered tissue tumor lysis buffer (1000 μl per 100 mg of tumor tissue, composition: 10 mM Tris buffer at pH 8.0, 137 mM NaCl, 1% Triton X-100, 10% glycerol, 10 μg/ml aprotinin, 10 μg/ml leupeptin, 1 mM PMSF, 0.4 mM orthovanadate) was added. The material was carefully mixed and transferred to Eppendorf tubes and allowed to thaw completely for 15 min. at 4°C. Subsequently the lysate was centrifuged for 15 min. at 20,000g and 4°C. After removal of the fat layer the supernatant was separated from the solid pellet material, split in aliquots and stored at -80°C until analysis. An aliquot was assessed by BCA-assay (Thermo Scientific #23223) giving protein concentrations in the range of 5–10 mg/ml.

Concentrations of mouse MIP-1alpha and MCP-1 were detected using the corresponding ELISA kits from R&D Systems (MMA00 and MJE00) according to the manufacturer’s instructions.

### *Ex vivo* analysis of tumor infiltrates

Explanted tumors were immediately transferred to RPMI1640-containing plates on ice and cut into 2–4mm pieces using scalpel. An enzymatic digestion was then performed during 30min at 37°C in medium containing Dispase II (1 mg/ml; Roche), Collagenase I (1mg/ml; Sigma) and DNAse I (0.01%; Roche). Samples were passed through a 70 μm-cell strainer, centrifuged, resuspended in medium and passed through a 40 μm-cell strainer. After one more washing step, cells were resuspended in FACS buffer (PBS containing 3% FCS and 2mM EDTA) and incubated with CD16/CD32 Fc Block (BD) before staining with the following specific antibodies (or their corresponding isotype controls): CD45-APC, CD11b-BV570, MHCII-FITC, CD163-PE, Ly6G-PerCP-Cy5.5, F4/80-PE-Cy7 and Ly6C-APC-Cy7, for 20min on ice (all antibodies from Biolegend). Finally, cells were washed and resuspended in FACS buffer containing 1 μg/ml DAPI and measured at a FACS Canto II (BD).

### Phagocytosis assays

For flow cytometry assays, mouse macrophages were generated by isolating bone marrow cells from healthy 5-week old C57BL/6 mice and culturing them on 10 cm UpCell plates (Nunc) for 7 days in DMEM medium (PAN Biotech) supplemented with 10% FCS (Gibco), 2mM L-Glutamine (PAA), Penicilin/Streptomycin (Roche), and 10 ng/ml recombinant mouse M-CSF (Biomol) before being activated for 24h with 100 ng/ml LPS (Sigma). Tumor cells were labeled with 2 μM CMFDA green (Life Technologies) for 1h before plating them together with the activated macrophages at a 1:2 ratio (tumor:macrophage) in the absence or presence of control or anti-CD44 antibodies at the given concentrations. Co-cultures were incubated for 30min and then cells were detached, washed and stained using an anti-CD11b BV570 antibody (Biolegend) to label the macrophages. DAPI was finally added for viable/dead gating, samples were measured at FACS Canto II and data analyzed using FlowJo software (TreeStar). A similar set of assays was performed using human macrophages, generated as stated below, yielding the same results. The phagocytic index (PI) was calculated according to the following formula: PI = (total number of engulfed cells/total number of counted macrophages) × (number of macrophages containing engulfed cells/total number of counted macrophages) × 100, where the total number of engulfed cells was calculated as the difference between the initial and final counts of tumor cells, and the CD11b^+^CMFDA^+^ double positive cells were considered as the number of macrophages containing engulfed cells. Two-way ANOVA was performed for statistical analysis and the Bonferroni’s method was used for multiple testing correction.

For live imaging, human peripheral blood mononuclear cells (PBMC) were isolated from whole blood by Ficoll centrifugation followed by negative isolation of human monocytes using the Monocyte Isolation kit II from Miltenyi. The obtained monocytes were stimulated with 20ng/ml rh-MCSF (Biomol) for 6 days in UpCell dishes (Nunc), then medium (RPMI-1640 without phenolred + 10% heat-inactivated FCS + 2mM L-glutamine + penicillin/streptomycin) was aspirated and fresh medium containing 20ng/ml rh-MCSF and 10ng/ml rh-IL-10 (RnD Systems) was added. After incubation for further 24h at 37°C, macrophages were detached, stained with 5 μM CMTMR Orange (Life Technologies), seeded at 1x10^4^ cells/well in 96er microtiter plates and further incubated at 37°C for 24h. Tumor cells were stained with 2μM CMFDA Green (Life Technologies) on assay day. After staining and detaching, target cells were incubated with 10μg/ml anti-CD44 antibody or huIgG1 isotype for 30min at RT. After two washing steps with PBS to remove excess antibody, cells were fixed with 4% paraformaldehyde for 15min at RT. Finally, 5x10^3^ tumor cells were pipetted to each well onto the macrophages. Plate was immediately spun down and images were recorded every 30 min for 10 hours using a Perkin Elmer Operetta HTS (a minimum of 15 fields/well/time point were recorded). The total number of CMFDA-positive cells was monitored using the Harmony software and statistically analyzed by Two-way ANOVA and the Bonferroni’s multiple comparison test.

## Supporting Information

S1 Fig Experimental setup for short-term MDA-MB231 xenograft in vivo study. (PPTX)

S2 Fig Functional analysis of gene-expression data. (A) Overview of biological processes and functions affected by anti-CD44 treatment RG7356 treatment in the tumor (left) and the mouse (right); (B) Heatmap view of predicted activity of biological processes obtained from IPA downstream processes analysis for the tumor (left) and the host (right). Activated processes are shown in different shades of orange, inhibited processes are shown in different shades of blue, if no activity assessment was possible, the biological process is shown in white.
(PPTX)

S3 Fig Overview of the top10 canonical pathways that are enriched at 8h of treatment in (A) the tumor and (B) the host.
(PPTX)

S4 Fig Venn-Diagram comparing the number of differentially expressed genes in the mouse (left) with the ones in the tumor (right).
(PPTX)

S5 Fig Network view of NFkB target genes as exported from IPA. (PPTX)

S6 Fig Statistical analysis of Luminex analytes changed in the tumor. Shown are the concentrations at different time points, during different treatments in the different strains.
(PPTX)

S7 Fig Statistical analysis of Luminex analytes changed in the host. Shown are the concentrations at different time points, during different treatments in the different strains.
(PPTX)

S8 Fig HCC-1937 shows no response to anti-CD44 treatment (18% TGI). (PPTX)

S1 Materials and Methods Initial Affymetrix experiments lead to immune response hypothesis. (DOCX)

S1 Table Biological Functions export–tumor. (XLS)

S2 Table Biological Functions export–host. (XLS)

S3 Table 8h CD44 treatment human genes. (XLS)

S4 Table 8h CD44 treatment mouse genes. (XLS)

S5 Table CD44-IgG4 treatment human genes. (XLS)

S6 Table CD44-IgG4 treatment mouse genes. (XLS)

S7 Table List of Luminex analytes. (XLSX)

S8 Table Statistical analysis of Luminex data. (XLSX)

S1 Video Phagocytosis under anti-CD44 treatment. (WMV)

S2 Video Phagocytosis under isotype control. (WMV)
