# Supplementary material for: Targeting Tumor Cells with Anti-CD44 Antibody Triggers Macrophage-Mediated Immune Modulatory Effects in a Cancer Xenograft Model
Source: PLoS One. 2016 Jul 27;11(7):e0159716. doi: 10.1371/journal.pone.0159716 (PMC4963023; doi:10.1371/journal.pone.0159716)
Supplement: S1 Fig — (PPTX) [file pone.0159716.s001.pptx]

## Slide 1
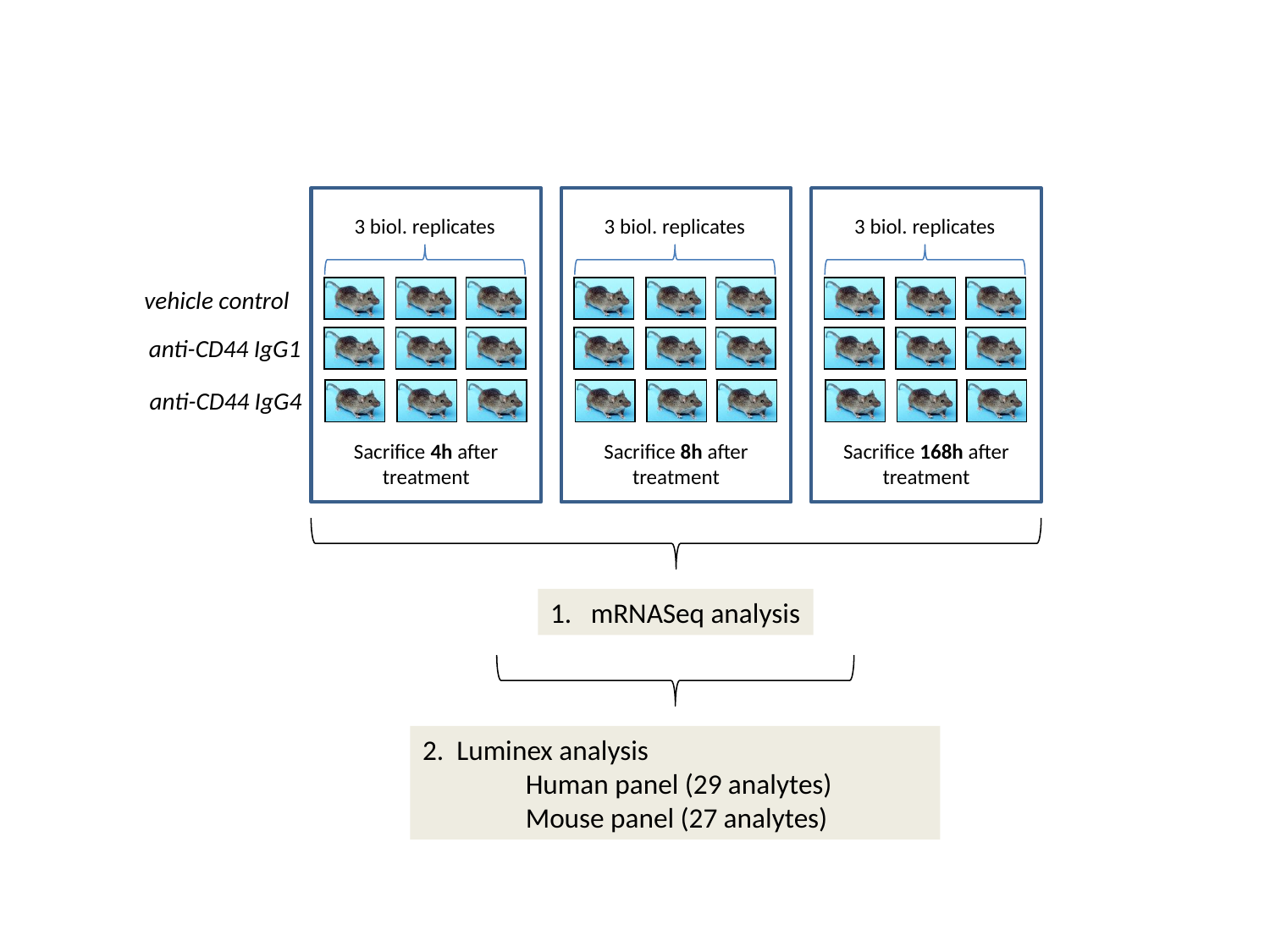

3 biol. replicates
3 biol. replicates
3 biol. replicates
vehicle control
anti-CD44 IgG1
anti-CD44 IgG4
Sacrifice 4h after treatment
Sacrifice 8h after treatment
Sacrifice 168h after treatment
1. mRNASeq analysis
2. Luminex analysis
Human panel (29 analytes)
Mouse panel (27 analytes)
