# Supplementary figures and images for: Targeting Tumor Cells with Anti-CD44 Antibody Triggers Macrophage-Mediated Immune Modulatory Effects in a Cancer Xenograft Model
Source: PLoS One. 2016 Jul 27;11(7):e0159716. doi: 10.1371/journal.pone.0159716 (PMC4963023; doi:10.1371/journal.pone.0159716)

## Slide 1
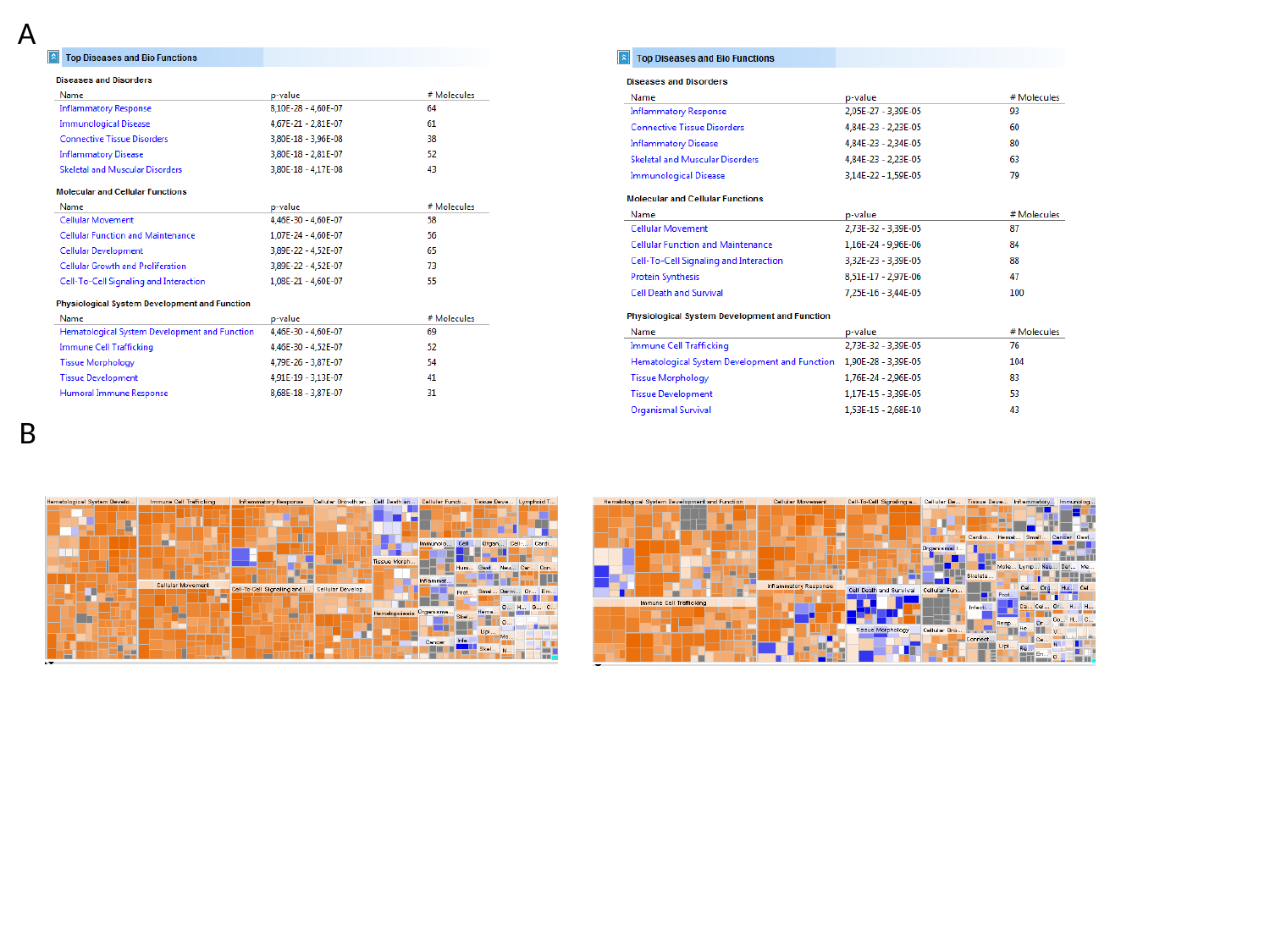

A
B

Supplement: S2 Fig — (A) Overview of biological processes and functions affected by anti-CD44 treatment RG7356 treatment in the tumor (left) and the mouse (right); (B) Heatmap view of predicted activity of biological processes obtained from IPA downstream processes analysis for the tumor (left) and the host (right). Activated processes are shown in different shades of orange, inhibited processes are shown in different shades of blue, if no activity assessment was possible, the biological process is shown in white. (PPTX) [file pone.0159716.s002.pptx]

## Slide 1
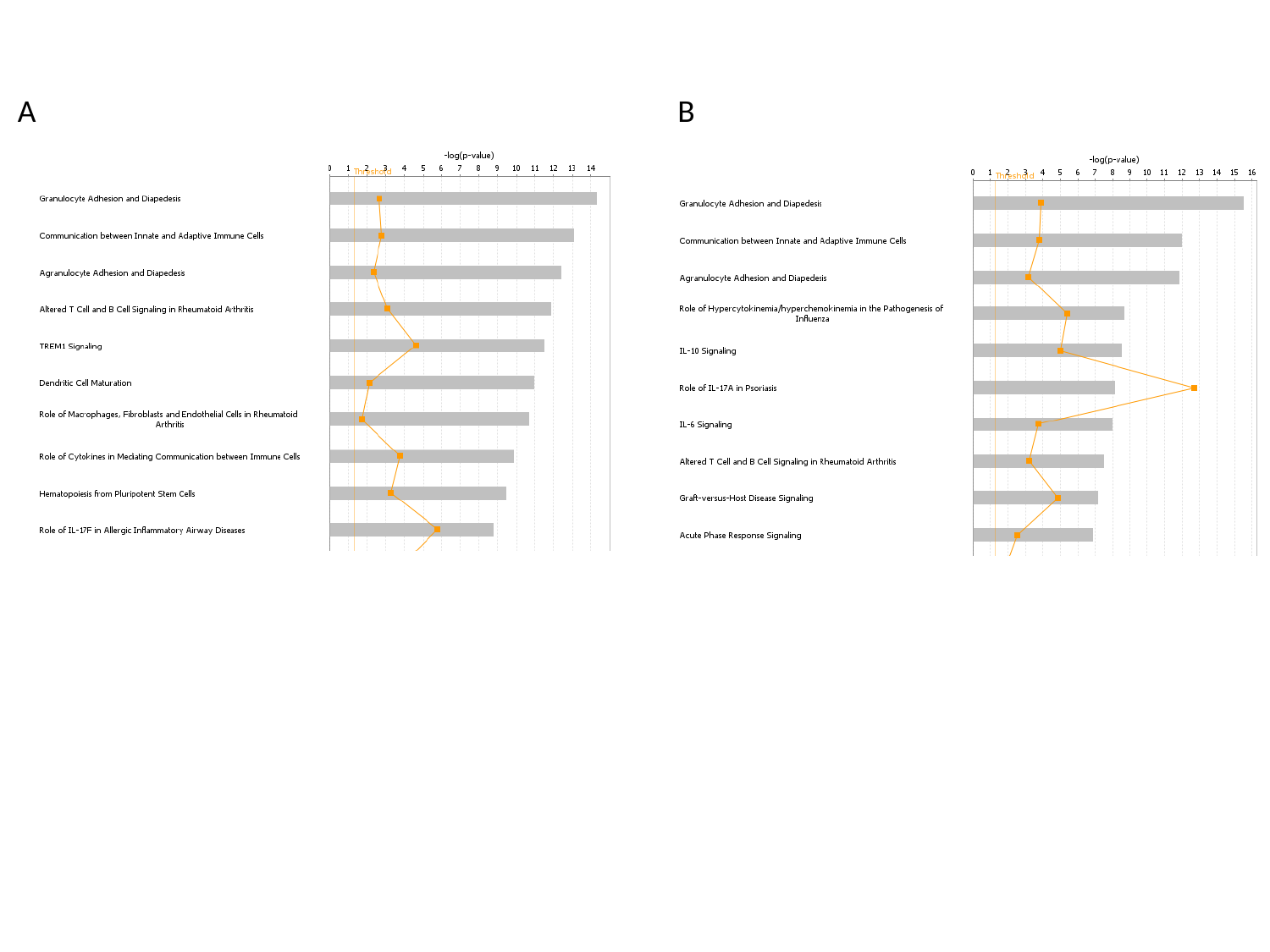

A
B

Supplement: S3 Fig — Overview of the top10 canonical pathways that are enriched at 8h of treatment in (A) the tumor and (B) the host. (PPTX) [file pone.0159716.s003.pptx]

## Slide 1
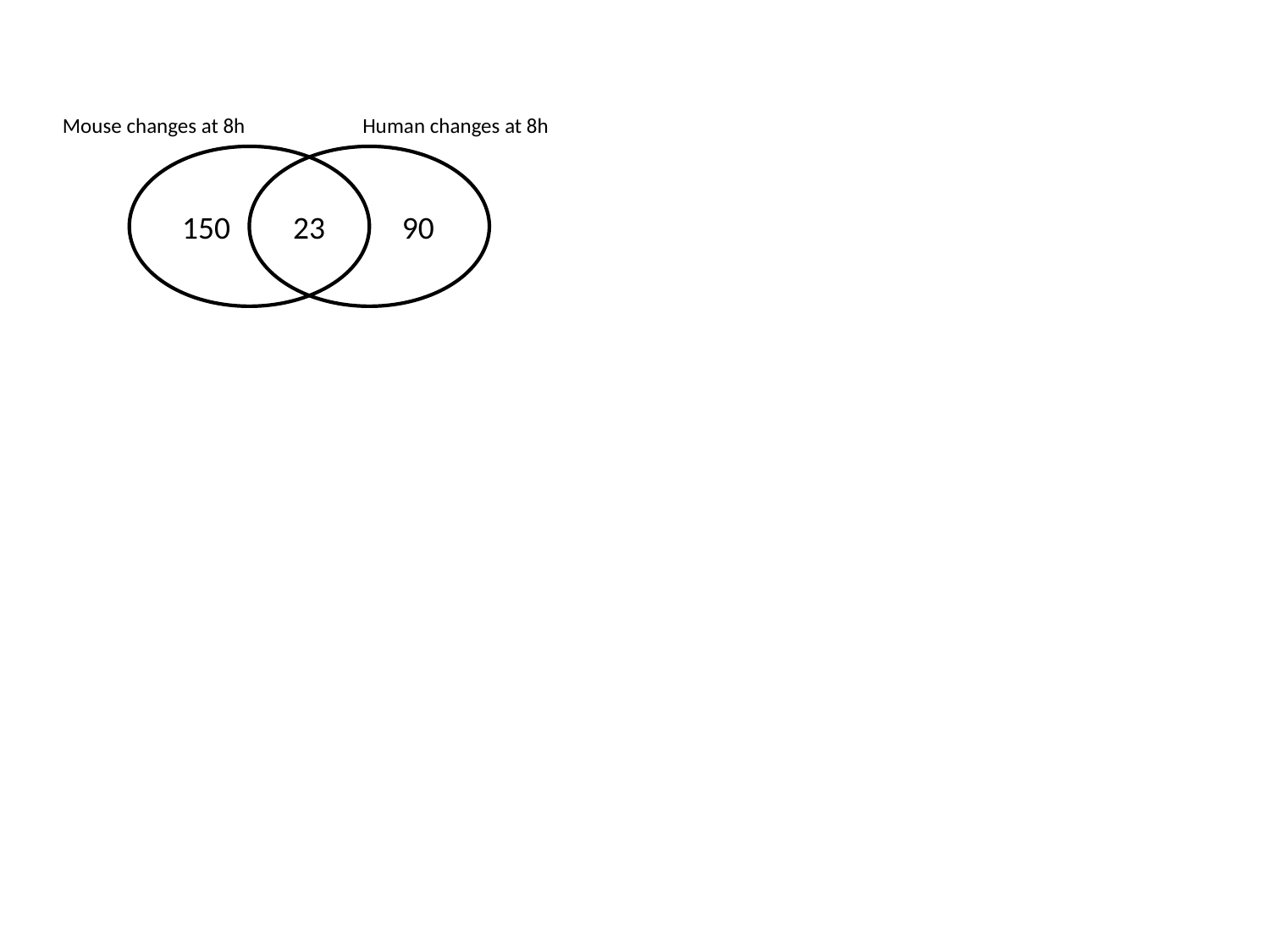

Mouse changes at 8h
Human changes at 8h
150
23
90

Supplement: S4 Fig — Venn-Diagram comparing the number of differentially expressed genes in the mouse (left) with the ones in the tumor (right). (PPTX) [file pone.0159716.s004.pptx]

## Slide 1
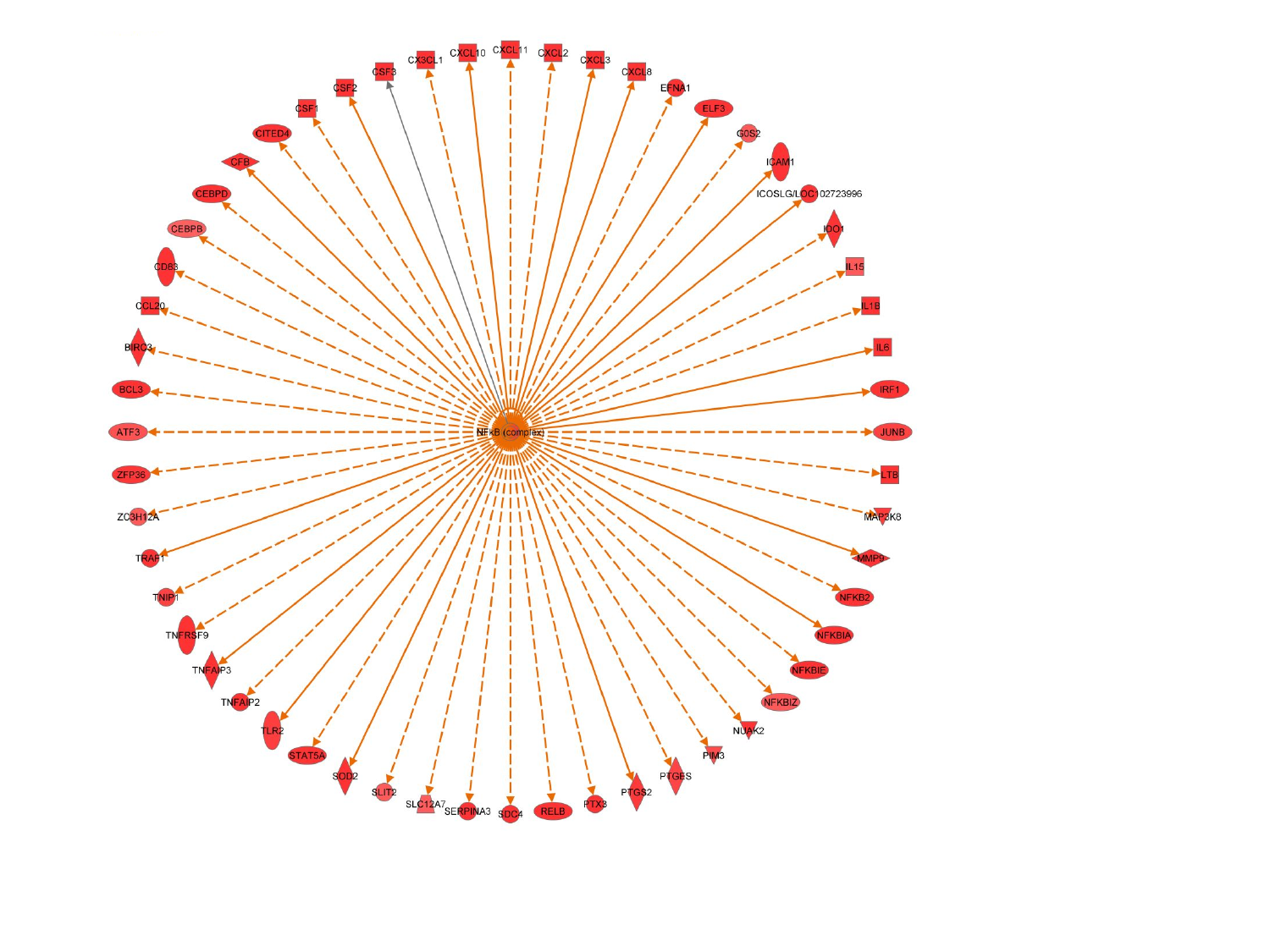

Supplement: S5 Fig — (PPTX) [file pone.0159716.s005.pptx]

## Slide 1
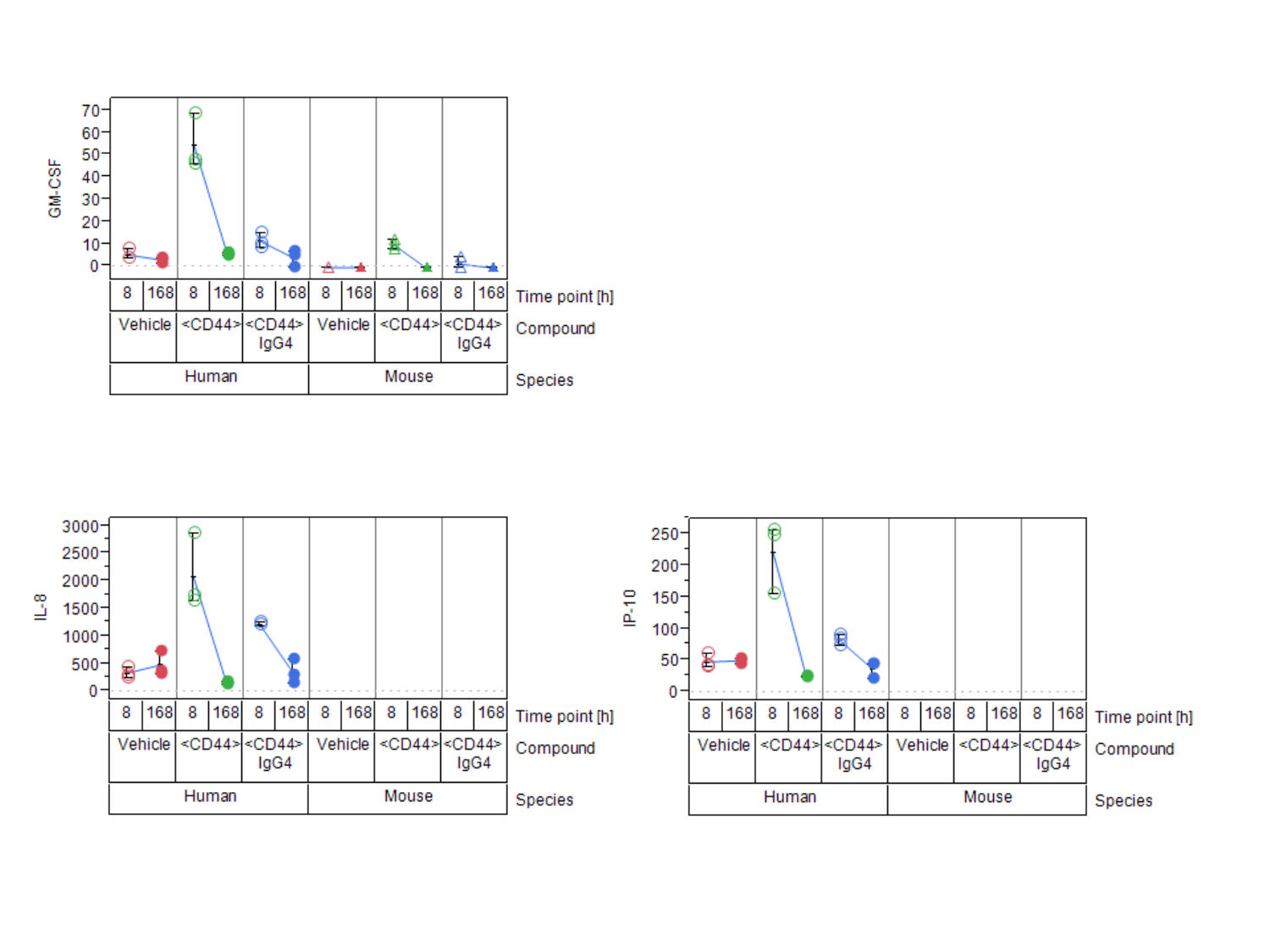

Supplement: S6 Fig — Shown are the concentrations at different time points, during different treatments in the different strains. (PPTX) [file pone.0159716.s006.pptx]
