# Supplementary material for: Targeting Tumor Cells with Anti-CD44 Antibody Triggers Macrophage-Mediated Immune Modulatory Effects in a Cancer Xenograft Model
Source: PLoS One. 2016 Jul 27;11(7):e0159716. doi: 10.1371/journal.pone.0159716 (PMC4963023; doi:10.1371/journal.pone.0159716)
Supplement: S7 Fig — Shown are the concentrations at different time points, during different treatments in the different strains. (PPTX) [file pone.0159716.s007.pptx]

## Slide 1
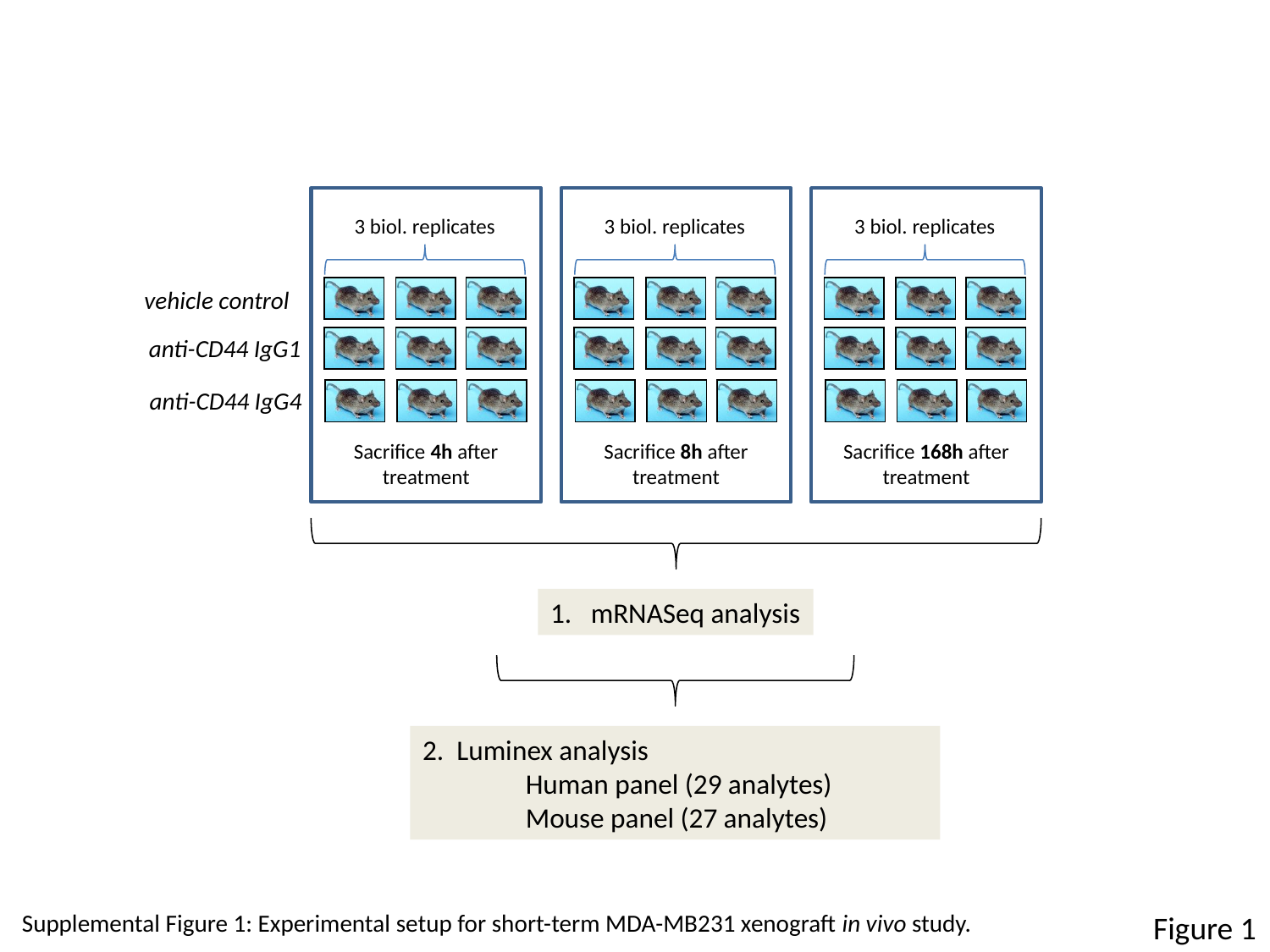

3 biol. replicates
3 biol. replicates
3 biol. replicates
vehicle control
anti-CD44 IgG1
anti-CD44 IgG4
Sacrifice 4h after treatment
Sacrifice 8h after treatment
Sacrifice 168h after treatment
1. mRNASeq analysis
2. Luminex analysis
Human panel (29 analytes)
Mouse panel (27 analytes)
Supplemental Figure 1: Experimental setup for short-term MDA-MB231 xenograft in vivo study.
Figure 1

## Slide 2
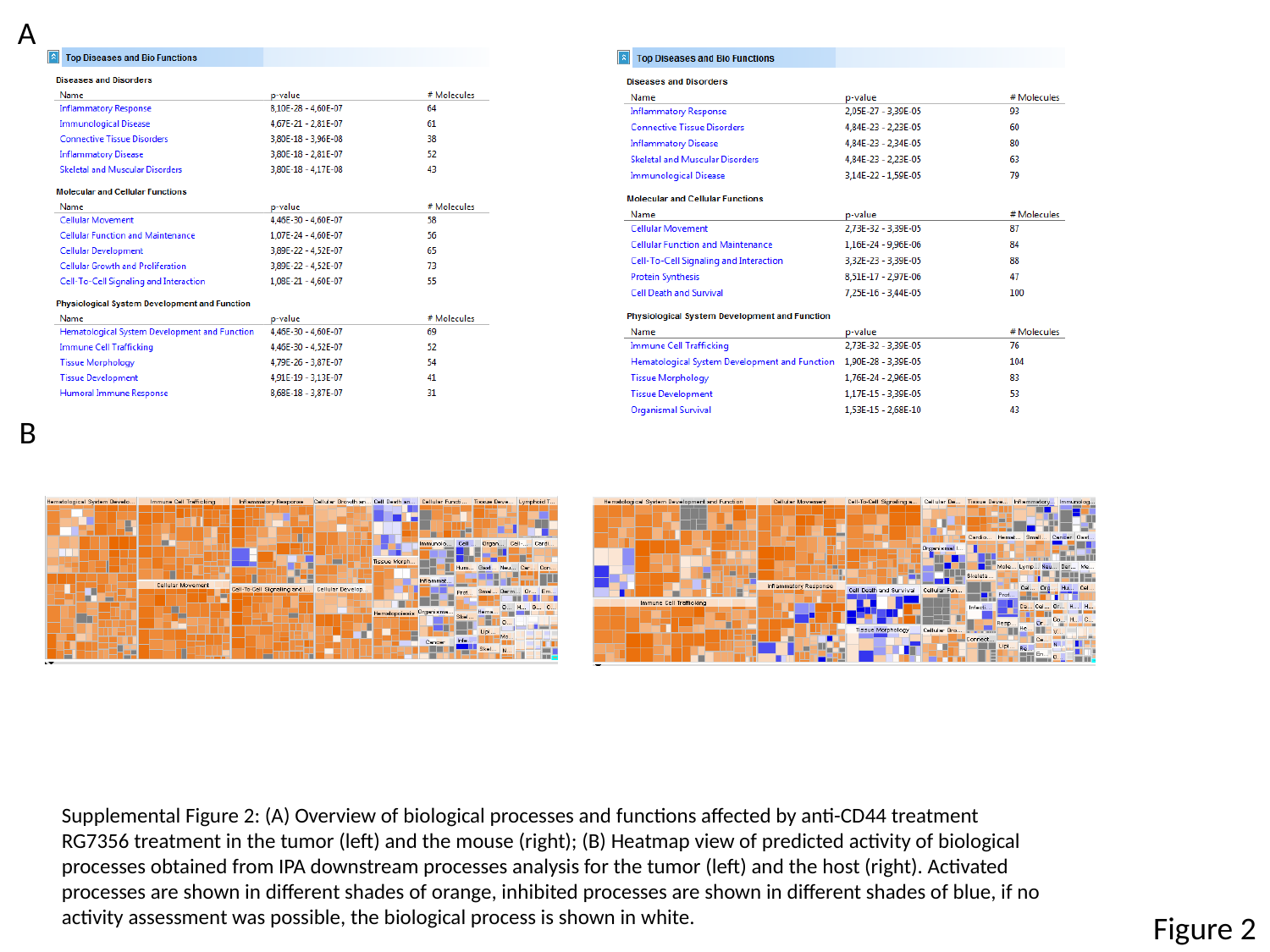

A
B
Supplemental Figure 2: (A) Overview of biological processes and functions affected by anti-CD44 treatment RG7356 treatment in the tumor (left) and the mouse (right); (B) Heatmap view of predicted activity of biological processes obtained from IPA downstream processes analysis for the tumor (left) and the host (right). Activated processes are shown in different shades of orange, inhibited processes are shown in different shades of blue, if no activity assessment was possible, the biological process is shown in white.
Figure 2

## Slide 3
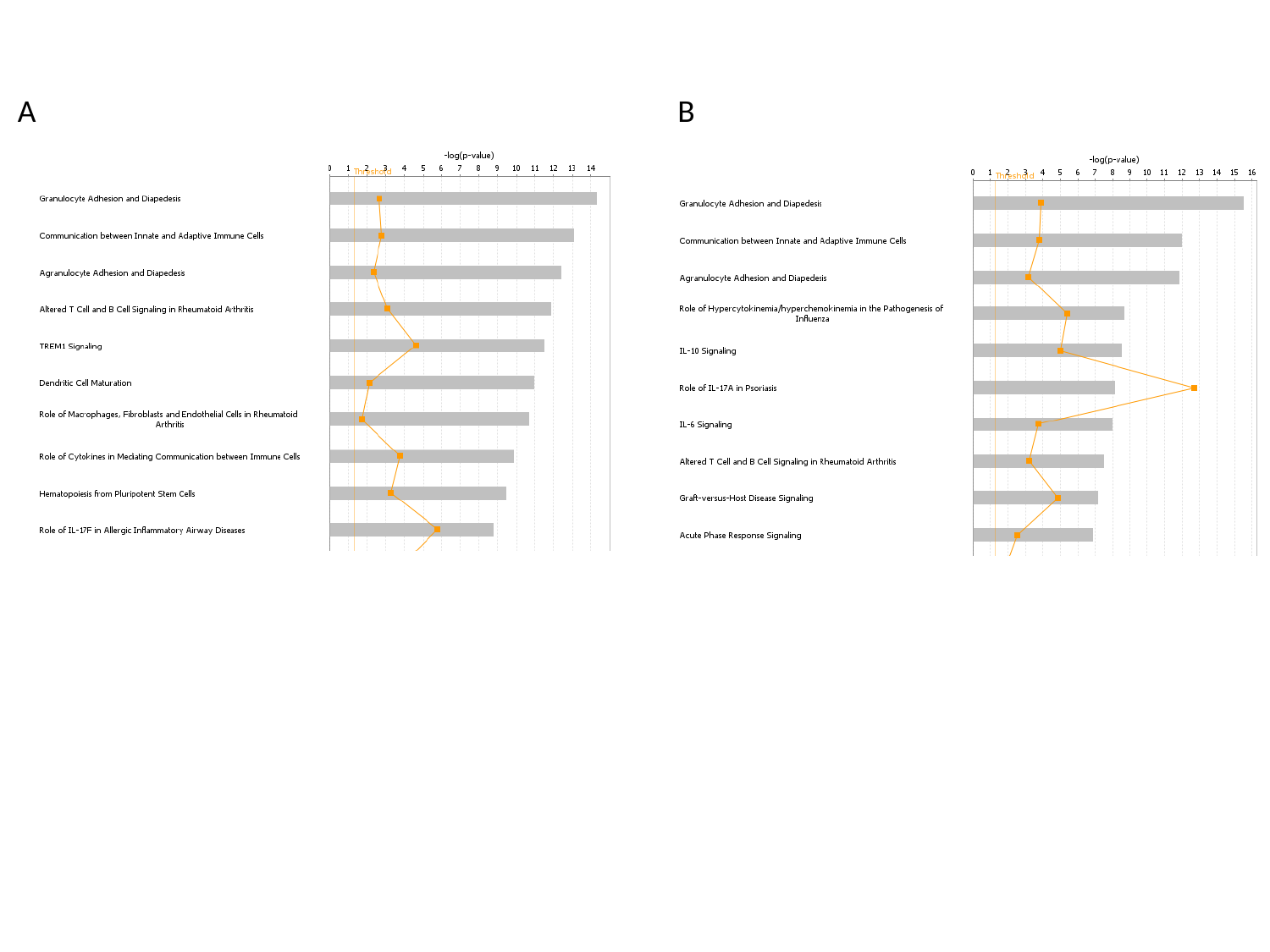

A
B

## Slide 4
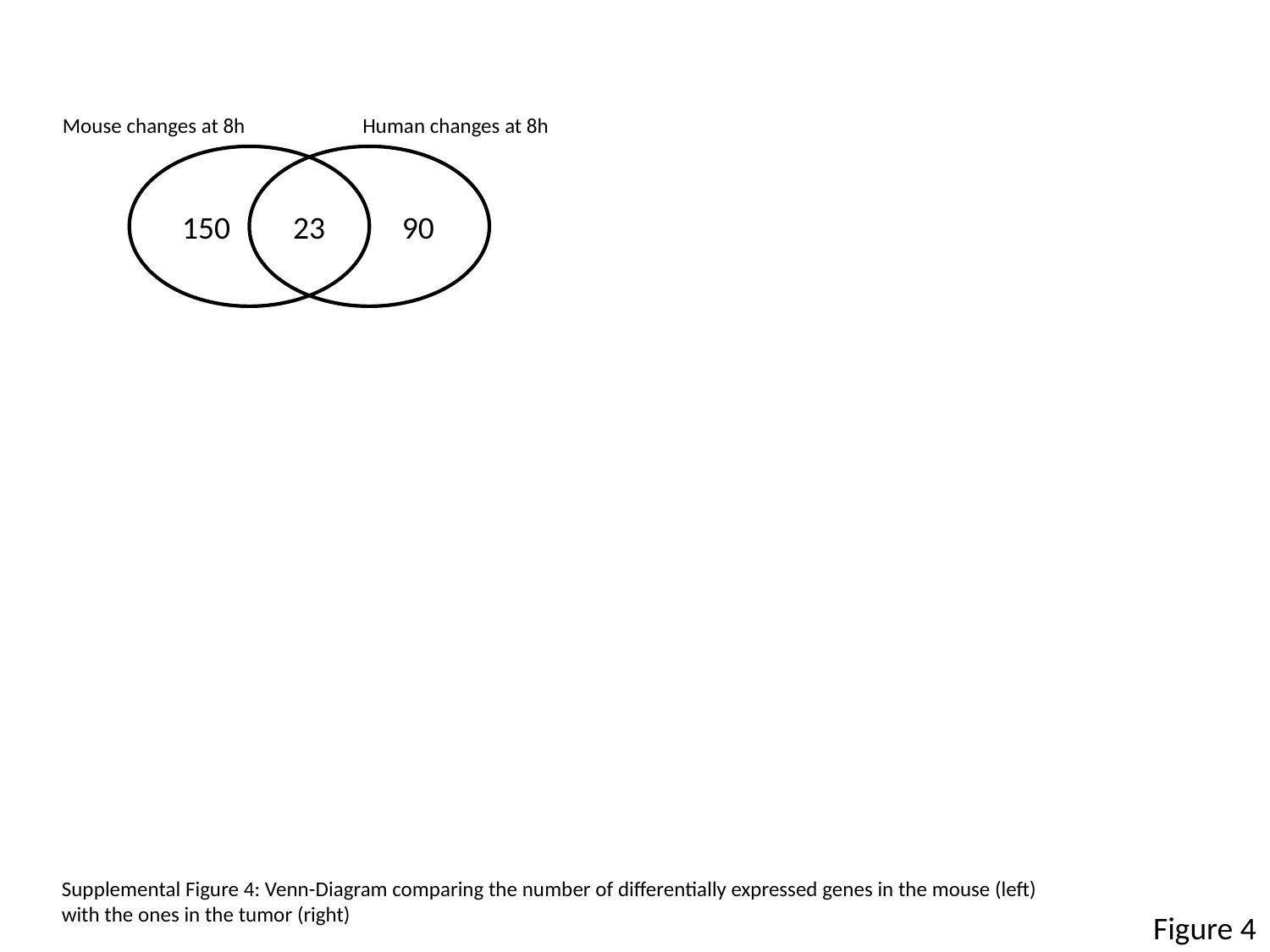

Mouse changes at 8h
Human changes at 8h
150
23
90
Supplemental Figure 4: Venn-Diagram comparing the number of differentially expressed genes in the mouse (left) with the ones in the tumor (right)
Figure 4

## Slide 5
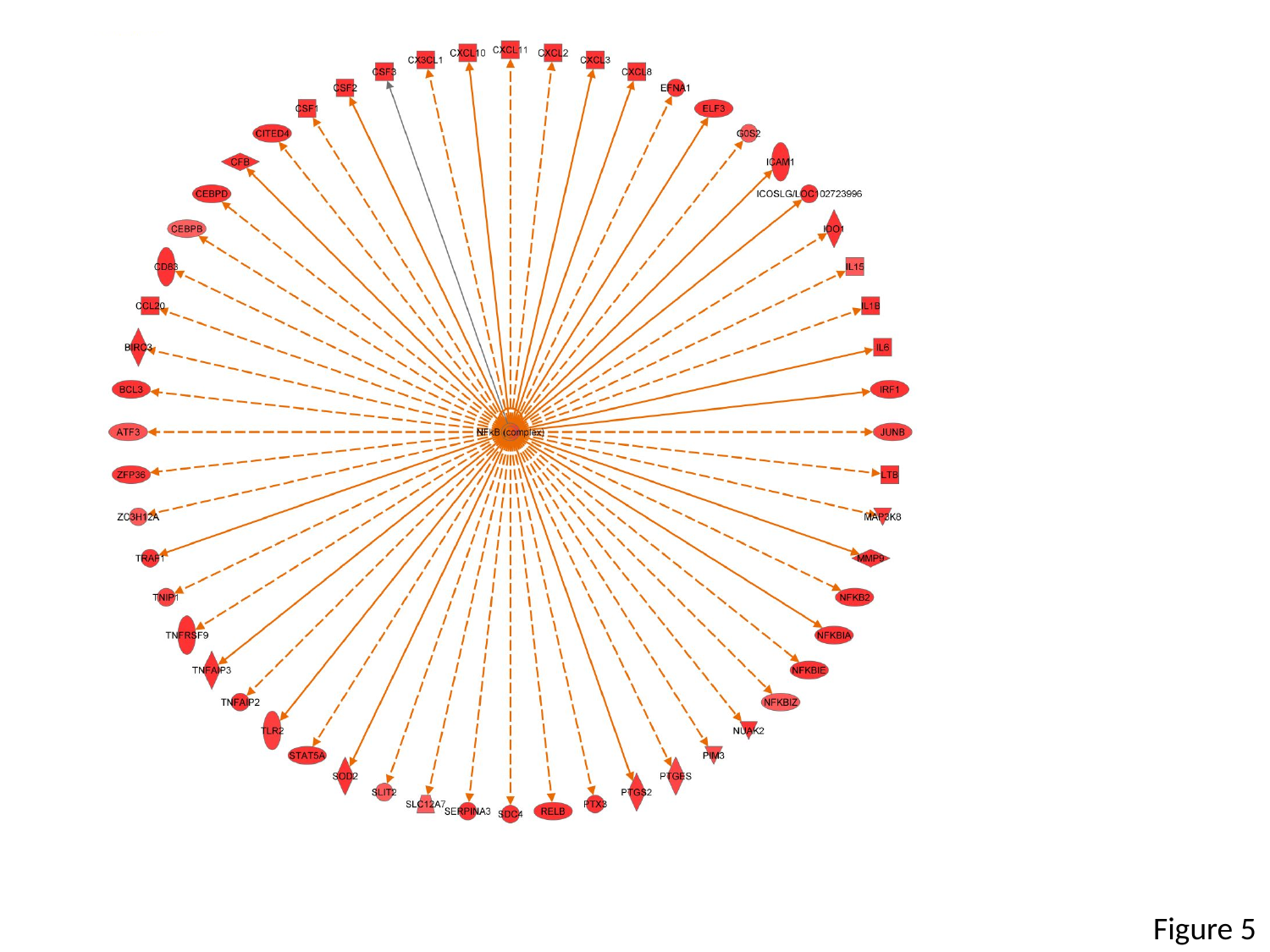

Figure 5

## Slide 6
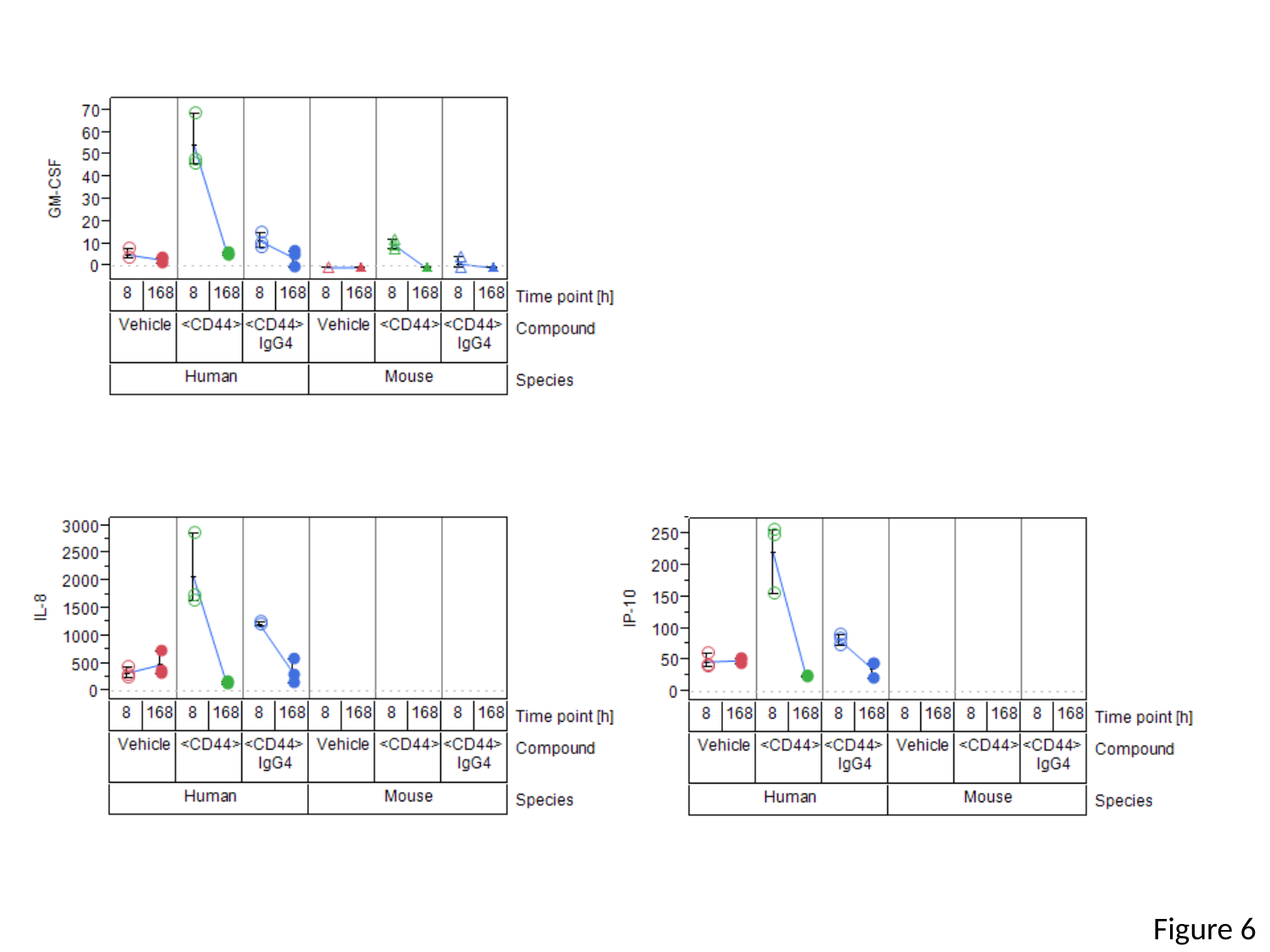

Figure 6

## Slide 7
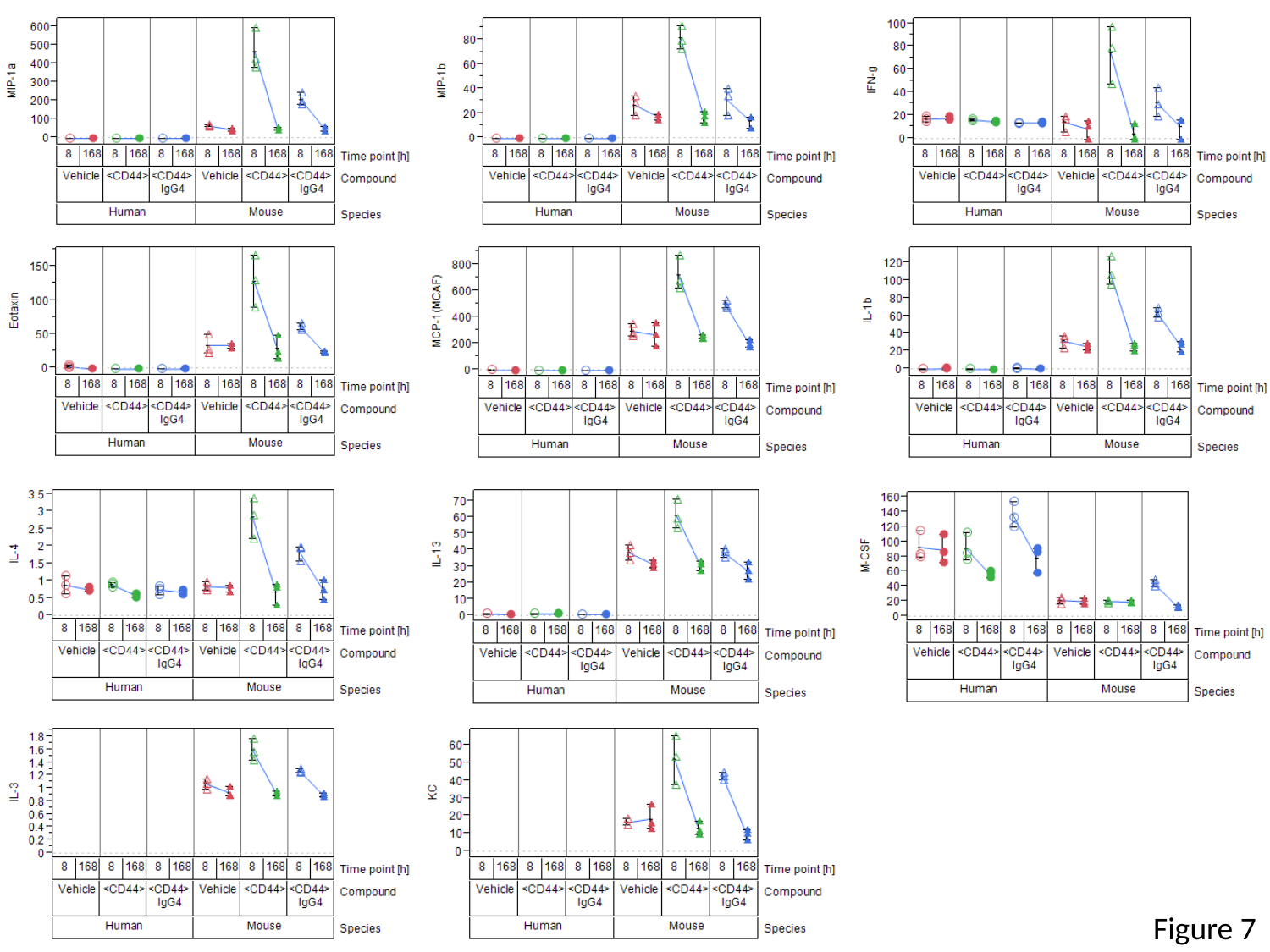

Figure 7

## Slide 8
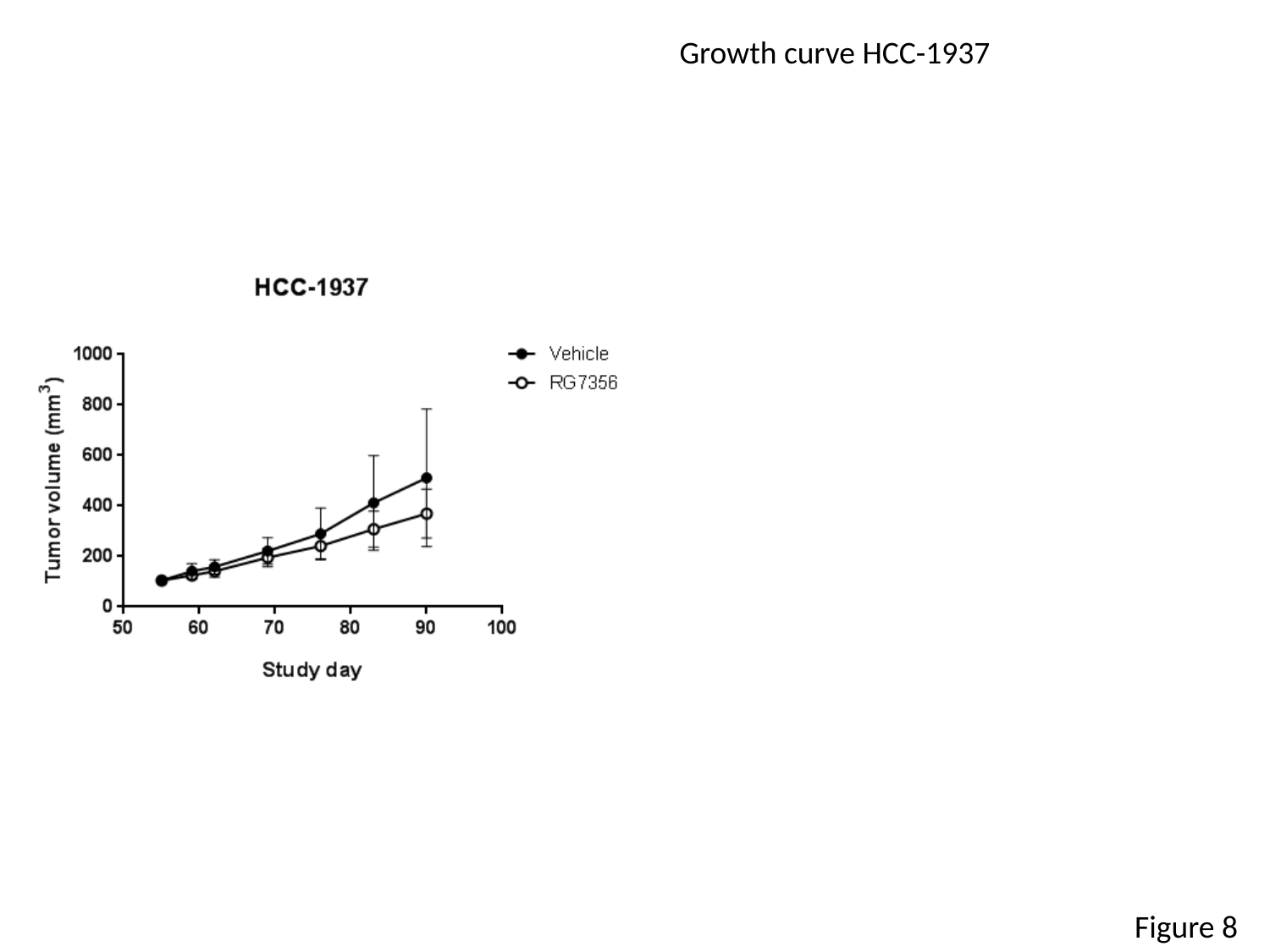

Growth curve HCC-1937
Figure 8
