# Supplementary material for: Targeting Tumor Cells with Anti-CD44 Antibody Triggers Macrophage-Mediated Immune Modulatory Effects in a Cancer Xenograft Model
Source: PLoS One. 2016 Jul 27;11(7):e0159716. doi: 10.1371/journal.pone.0159716 (PMC4963023; doi:10.1371/journal.pone.0159716)
Supplement: S1 Materials and Methods — (DOCX) [file pone.0159716.s009.docx]

Supplemental Material and Methods

**A combined gene-expression and phosphoproteomic analysis reveals significant inflammatory response in the tumor.**

Using global phosphoproteome analysis, we have recently shown a modulation of the MAPK pathway upon treatment with RG7356 (1). To understand how and to what extend the observed phosphoproteomic changes translate into differential gene expression we conducted a gene-expression profiling experiment of the same xenograft *in vivo* model MDA-MB-231. Here, we used an Affymetrix HG-U133Plus2 microarray to profile the RG7356-treated tumors in a time-course experiment (4h, 8h, 24h and 168h), in order to analyze the kinetics of expression of target genes upon treatment. Differential gene-expression analysis showed significant changes after 4 and 8 hours, while at the later time points no relevant changes occurred (Figure 1).


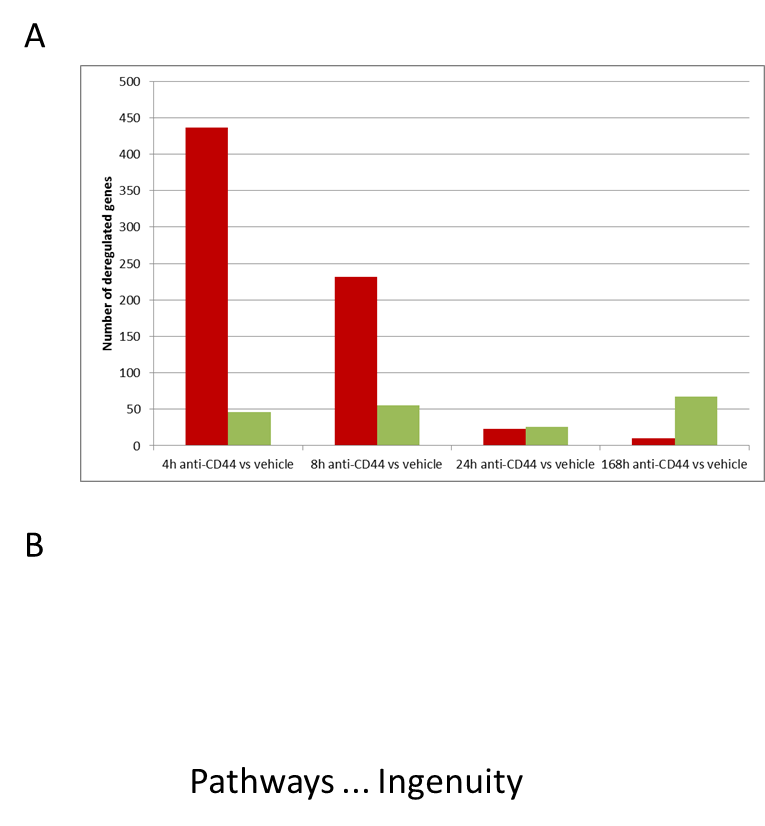


***Figure 1:*** *Number of differentially expressed transcripts at 4 different timepoints (4hrs, 8hrs, 24hrs, 168hrs) measured on the HG-U133Plus2 array. For each timepoint, the expression under anti-CD44 treatment was compared to the vehicle control. Up-regulated genes are shown in red, down-regulated genes are shown in green.*

Therefore, we focused our investigation on the acute setting and further analyzed the obtained results at early time points using Ingenuity Pathway Analysis (IPA; www.ingenuity.com). The outcome revealed differential regulation of several pathways involved in the immune response (data not shown). We then utilized Selventa’s causal reasoning approach to derive upstream regulators that can explainn the observed gene-expression changes (2). In short, Selventa uses a database of literature-derived signatures which consist of an upstream regulator, the genes regulated by it as well as the direction of regulation. Depending on the amount and direction of regulated genes in the dataset, it is possible to make a call about a specific upstream regulator being activated or inhibited. These are then incorporated into a network of regulators to support the generation of a hypothesis that can explain the observed changes. To supplement the gene-expression findings, we incorporated the phosphoproteomics data from our previous experiment (1) into the analysis. Figure 2A shows the underlying evidence for the most prominent hypothesis that anti-CD44 treatment leads to the activation of an anti-tumor immune response.


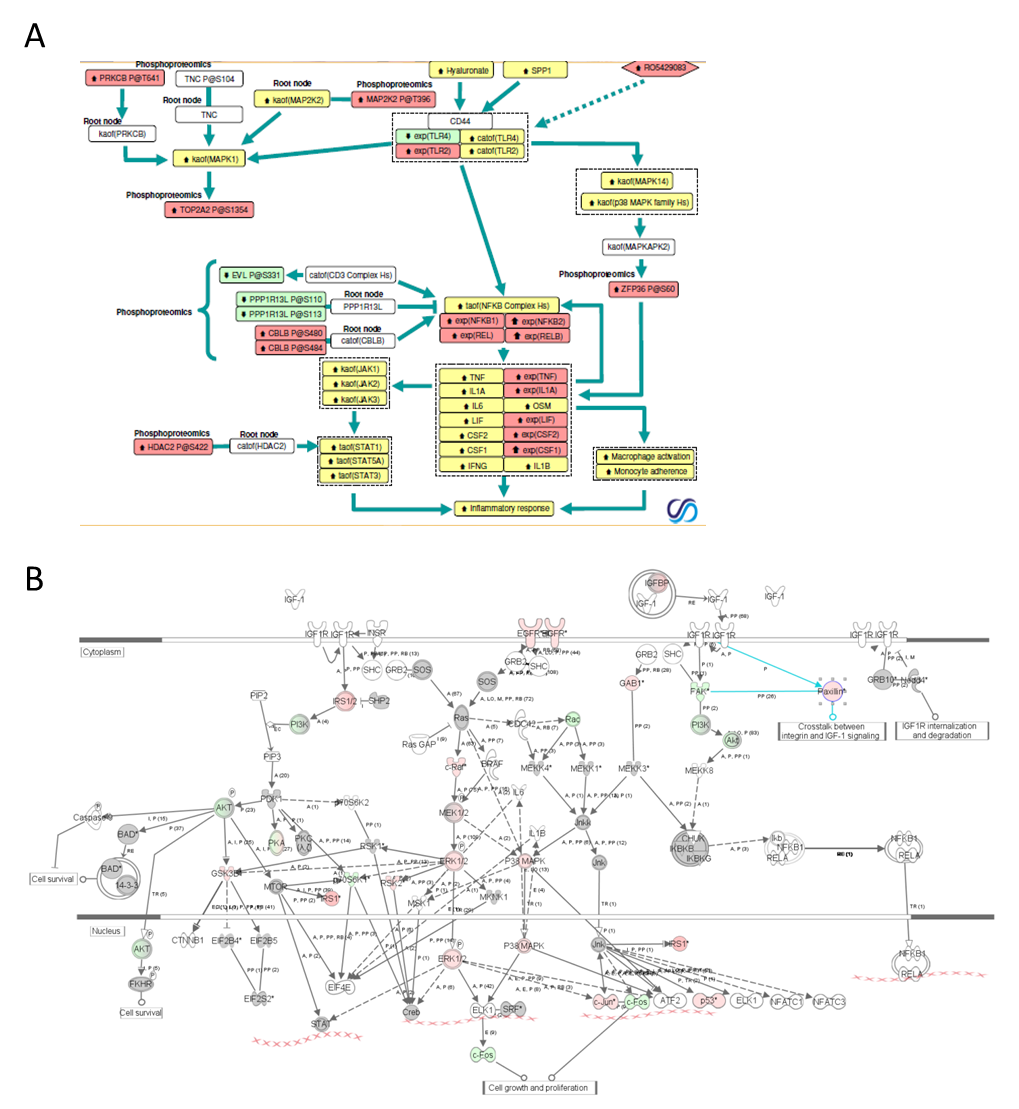


***Figure 2:*** *(A) Pathway map as a result of Selventa’s reverse causal reasoning approach (B) Extended view of MAPK signaling (pathway map from IPA) overlayed with phosphoproteomics results.*

These findings are in line with our recent observations on the modulation of the MAPK pathway upon treatment with RG7356 (1). The activation of the MAPK pathway and downstream targets after 0.5h or 1.5h (Figure 2B) supports the initiation and prolongation of an immune response after 4h and 8h of treatment seen on the gene expression level.

Taken together, the combined gene-expression and phospho-proteomics analyses suggest the activation of the immune system in this xenograft model in response to treatment with an anti-CD44 antibody.

Reference List

(1) Weigand S, Herting F, Maisel D, Nopora A, Voss E, Schaab C, et al. Global quantitative phosphoproteome analysis of human tumor xenografts treated with a CD44 antagonist. Cancer Res 2012 Sep 1;72(17):4329-39.

(2) Laifenfeld D, Qiu L, Swiss R, Park J, Macoritto M, Will Y, et al. Utilization of causal reasoning of hepatic gene expression in rats to identify molecular pathways of idiosyncratic drug-induced liver injury. Toxicol Sci 2014 Jan;137(1):234-48.
